# Short-term thermal photosynthetic responses of C_4_ grasses are independent of the biochemical subtype

**DOI:** 10.1093/jxb/erx350

**Published:** 2017-10-16

**Authors:** Balasaheb V Sonawane, Robert E Sharwood, Susanne von Caemmerer, Spencer M Whitney, Oula Ghannoum

**Affiliations:** 1 ARC Centre of Excellence for Translational Photosynthesis and Hawkesbury Institute for the Environment, Western Sydney University, Richmond NSW, Australia; 2 ARC Centre of Excellence for Translational Photosynthesis and Research School of Biology, Australian National University, Canberra ACT, Australia

**Keywords:** Biochemical subtypes, C_4_ photosynthesis, CO_2_ concentrating mechanism, thermal responses

## Abstract

C_4_ photosynthesis evolved independently many times, resulting in multiple biochemical pathways; however, little is known about how these different pathways respond to temperature. We investigated the photosynthetic responses of eight C_4_ grasses belonging to three biochemical subtypes (NADP-ME, PEP-CK, and NAD-ME) to four leaf temperatures (18, 25, 32, and 40 °C). We also explored whether the biochemical subtype influences the thermal responses of (i) *in vitro* PEPC (*V*_pmax_) and Rubisco (*V*_cmax_) maximal activities, (ii) initial slope (IS) and CO_2_-saturated rate (CSR) derived from the *A*-*C*_i_ curves, and (iii) CO_2_ leakage out of the bundle sheath estimated from carbon isotope discrimination. We focussed on leakiness and the two carboxylases because they determine the coordination of the CO_2_-concentrating mechanism and are important for parameterizing the semi-mechanistic C_4_ photosynthesis model. We found that the thermal responses of *V*_pmax_ and *V*_cmax_, IS, CSR, and leakiness varied among the C_4_ species independently of the biochemical subtype. No correlation was observed between *V*_pmax_ and IS or between *V*_cmax_ and CSR; while the ratios *V*_pmax_/*V*_cmax_ and IS/CSR did not correlate with leakiness among the C_4_ grasses. Determining mesophyll and bundle sheath conductances in diverse C_4_ grasses is required to further elucidate how C_4_ photosynthesis responds to temperature.

## Teaser

*In vitro* and *in vivo* thermal photosynthetic responses vary among C_4_-grasses independently of biochemical subtype, and leakiness does not correlate with PEPC/Rubisco or initial slope/CO_2_-saturated photosynthesis rate.

## Introduction

Understanding how photosynthesis responds to temperature is critical to our ability to predict the responses of natural and cropping ecosystems to climate change. Modelling the photosynthetic responses of C_3_ plants to temperature is a routine task where we have a growing appreciation of the natural variation in the underlying mechanisms, such as the temperature dependence of mesophyll conductance (*g*_m_) and the kinetics of the CO_2_-fixing enzyme ribulose 1,5-bisphosphate carboxylase/oxygenase (Rubisco) (Farquhar *et al.*, 2001; Bernacchi *et al.*, 2013; Walker *et al.*, 2013; von Caemmerer and Evans, 2015; Sharwood *et al.*, 2016*a*). Unlike its C_3_ counterpart, the semi-mechanistic C_4_ photosynthesis model (Farquhar, 1983; von Caemmerer, 2000) has not been comprehensively tested across a wide range of species and temperatures. Addressing this gap is critical given that C_4_ plants include some of the world’s most important food, feed, and biofuel crops (e.g. maize, sorghum, and sugarcane), dominate the understory of warm-climate grasslands and savannas (Ehleringer and Monson, 1993; Brown, 1999; Sage *et al.*, 2013), and account for 20–25% of terrestrial productivity (Lloyd and Farquhar, 1994).

C_4_ photosynthesis has evolved independently many times, resulting in multiple biochemical pathways named after the primary C_4_-acid decarboxylase enzyme found in the bundle sheath cells (BSCs), which are NADP-malic enzyme (-ME), NAD-ME, and phosphoenolpyruvate carboxykinase (PEP-CK) (Hatch 1987). Although PEP-CK operates as a secondary decarboxylase in many C_4_ species (Leegood and Walker, 2003; Furbank, 2011; Sharwood *et al.*, 2014; Wang *et al.*, 2014), the primary decarboxylase is generally associated with a suite of anatomical, biochemical, and physiological features (Gutierrez *et al.*, 1974; Hattersley, 1992; Kanai and Edwards, 1999; Ghannoum *et al.*, 2011). The grass family includes species from all biochemical subtypes, but little is known about how these different pathways respond to temperature.

C_4_ photosynthesis is characterized by the operation of a CO_2_-concentrating mechanism (CCM) comprising a C_4_ cycle that fixes atmospheric CO_2_ into a C_4_ acid in mesophyll cells (MCs) that is then transported to, and decarboxylated in, BSCs where Rubisco and the C_3_ cycle are localized (Hatch, 1987). The C_4_ cycle is faster than the C_3_ cycle, resulting in elevated CO_2_ in the BSCs and thus minimizing photorespiration and maximizing CO_2_ assimilation rates at low intercellular CO_2_ (*C*_i_) (Hatch, 1987; Kanai and Edwards, 1999). Some CO_2_ leaks out of the BSCs into the surrounding MCs, costing additional ATP that is required for the phosphorylation of phospenolpyruvate (PEP) in the MCs (Hatch, 1987).

Leakiness (ϕ) describes the efficiency of C_4_ photosynthesis and is defined as the rate of CO_2_ leakage out of the BSCs as a fraction of the rate of PEP carboxylation or C_4_ cycle rate (*V*_p_) (Farquhar, 1983). According to the C_4_ model, CO_2_ leakage (*L*) depends on the BSC wall conductance to CO_2_ (*g*_bs_) and the gradient between the CO_2_ concentration in the BSCs (*C*_bs_) and MCs (*C*_m_), such that *L*=*g*_bs_(*C*_bs_–*C*_m_). In turn, the BSC–MC CO_2_ gradient depends on the balance between the activity of the C_4_ (e.g. PEP carboxylase, PEPC) and C_3_ (e.g. Rubisco) cycles (Henderson *et al.*, 1992; von Caemmerer, 2000). The C_4_ photosynthesis model stipulates that the initial slope of the *A*-*C*_i_ curve (IS) depends on the maximal PEPC activity (*V*_pmax_) while the CO_2_-saturated rate (CSR) depends on maximal Rubisco activity (*V*_cmax_), in addition to other factors (von Caemmerer, 2000). Consequently, the thermal responses of *V*_pmax_ and *V*_cmax_ are expected to influence the thermal response of both IS and CSR as well as the BSC–MC CO_2_ gradient, and hence leakiness. Therefore, in this study we asked whether the thermal responses of these key parameters (*V*_pmax_, *V*_cmax_, IS, CSR, and ϕ) depend on the biochemical subtype of the C_4_ species.

In a recent study, Sharwood *et al.* (2016*a*) showed that the declining response of Rubisco’s *in vitro* CO_2_/O_2_ specificity (*S*_c/o_) with increasing temperature was more pronounced for NAD-ME relative to PEP-CK and NADP-ME species. Given that the CSR of C_4_ leaves depends strongly on Rubisco and its catalytic properties, we predict that, relative to the other two subtypes, *V*_cmax_ of NAD-ME species may increase less with temperature, with possible consequences on *V*_pmax_/*V*_cmax_ and ϕ. These predictions remain untested, especially in that thermal responses of *V*_pmax_ have been less widely studied than *V*_cmax_ (Tieszen and Sigurdson, 1973; Pittermann and Sage, 2000; Sage, 2002; Boyd *et al.*, 2015; Sharwood *et al.*, 2016*a*).

In an earlier study, high leaf temperature was found to increase O_2_ uptake in NAD-ME but not NADP-ME species, pointing to increased Mehler reaction and/or Rubisco oxygenation at higher temperature in NAD-ME species (Siebke *et al.*, 2003). Higher Rubisco oxygenation may be symptomatic of a less favorable CO_2_/O_2_ concentration ratio in the BSCs, which is corroborated by the less relaxed Rubisco affinity for CO_2_ (higher *K*_m(CO2)_) in NAD-ME relative to NADP-ME subtypes (Sharwood *et al.*, 2016*a*). The suberin lamella lining the BSC walls of NADP-ME and PEP-CK grasses may reduce *g*_bs_, which could impact *C*_bs_ (Hatch, 1987; von Caemmerer and Furbank, 2003). Consequently, we predict that *C*_bs_ and hence leakiness may be affected differently by temperature depending on the C_4_ subtypes. Previous measurements revealed a slight decrease in leakiness in response to increasing temperature between 21 and 35 °C for the C_4_ monocot *Sorghum bicolor* (Henderson *et al.*, 1992). To our knowledge, the thermal response of leakiness has not yet been compared in C_4_ grasses with different biochemical subtypes.

The temperature dependence of key parameters of the C_4_ photosynthesis model has primarily been determined for a few representative species, such as maize and *Setaria* (Henderson *et al.*, 1992; Chen *et al.*, 1994; Massad *et al.*, 2007; Boyd *et al.*, 2015). In addition, parameterization can be done *in vivo* (derived from gas exchange) or *in vitro* (derived from enzyme assays), but comparisons between these two parameterization methods remains unexplored for different C_4_ species and temperatures. Relationships between ϕ and the ratio of *in vivo* (IS/CSR) or *in vitro* (*V*_pmax_/*V*_cmax_) activities of C_4_ and C_3_ carboxylases have been demonstrated for single species under different growth environments (Ranjith *et al.*, 1995; Saliendra *et al.*, 1996; Meinzer and Zhu, 1998; Gong *et al.*, 2017). However, such relationships have rarely been explored for diverse C_4_ grasses at different temperatures. Addressing these knowledge gaps is critical to our ability to interpret gas exchange data and relate them to underlying biochemistry, as has been done for C_3_ plants (von Caemmerer and Farquhar, 1981; Hudson *et al.*, 1992; Bernacchi *et al.*, 2001).

Consequently, in this study we measured the photosynthetic thermal responses of eight diverse C_4_ grasses belonging to the three biochemical subtypes. We aimed at determining whether the C_4_ biochemical subtype influences the thermal responses of (i) *in vitro* PEPC (*V*_pmax_) and Rubisco (*V*_cmax_) maximal activities, (ii) initial slope (IS) and CO_2_-saturated rate (CSR) derived from the *A*-*C*_i_ curves, and (iii) CO_2_ leakage out of the bundle sheath as estimated from carbon isotope discrimination. Our results showed that the thermal photosynthetic responses varied among the C_4_ species independently of the biochemical subtype. We also observed that the ratios of IS/CS and *V*_pmax_/*V*_cmax_ were uncorrelated with each other or with leakiness when determined for a range of C_4_ species. Finally, we derived constants for thermal dependency that can be incorporated in the C_4_ photosynthesis model.

## Material and methods

### Plant culture

The experiment was conducted in a naturally lit glasshouse chamber (5 m^3^) with a mean noon photosynthetic photon flux density (PPFD) of 1230 ± 22 µmol quanta m^−2^ s^−2^ (±SE) and a 10-h photoperiod (see Supplementary Fig. S1 at *JXB* online). The air temperature inside the glasshouse compartment was regulated and day/night temperatures averaged 28/22 °C. Relative humidity was monitored and ranged between 60–80% during the day. Grass seeds (Table 1) were obtained from the Australian Plant Genetic Resources Information System, Australia. Seeds were sown in germination trays containing a common germination mixture. Seedlings at 3–4 weeks old were transplanted into the experimental pots (2 l) containing Osmocote^®^ Professional – Seed Raising & Cutting Mix (Scotts; www.scottsaustralia.com.au). Nutrients were supplied through the addition of Osmocote^®^ Plus Trace Element: Total All Purpose (N:P:K=19.4:1.6:5) (Scotts) and periodic watering with soluble Aquasol (N:P:K=23.3:3.95:14) (Yates; www.yates.com.au/). For each species there were eight pots, each of which contained a single plant. Pots were well-watered and regularly rotated within the glasshouse chamber.

**Table 1.** List of C_4_ grasses used in the current study.

C_4_ Subtype	C_4_ tribe	Species
NADP-ME	Paniceae Andropogoneae	*Cenchrus ciliaris* *Sorghum bicolor* *Zea mays*
PEP-CK	Paniceae Chloridoideae	*Eriochloa meyeriana* *Megathyrsus maximus* *Chloris gayana*
NAD-ME	Paniceae Chloridoideae	*Panicum coloratum* *Leptochloa fusca*

### Leaf gas exchange measurements

Leaf gas exchange measurements were carried out using a portable open photosynthesis system (LI-6400XT, LI-COR, Lincoln, USA). Measurements were conducted between 10.00 h and 14.00 h, 7–8 weeks after transplanting, on the last fully expanded leaf (LFEL) attached on the main stem. Prior to gas exchange measurements, plants were moved to another chamber where air temperature could be changed separately. Measurements were made at leaf temperatures of 18, 25, 34, and 40 °C by using the internal heating system of the photosynthesis unit in conjunction with the glasshouse chamber heating system, whilst maintaining a relatively constant humidity inside the leaf chamber. Prior to measurements, each leaf was allowed to reach a steady state of CO_2_ uptake at ambient CO_2_ (Reference=400 μl l^−1^), PPFD of 1800 μmol m^−2^ s^−1^, and relative humidity of 50–70%. A steady-state measurement was taken at each of the four leaf temperatures, and this was followed by measuring the responses of CO_2_ assimilation rate (*A*) to step increases of intercellular CO_2_ (*C*_i_) by raising the LI-6400XT leaf chamber [CO_2_] in 10 steps (i.e. 50, 100, 150, 200, 250, 325, 400, 650, 1200, and 1500 μl l^−1^) with 2 and 3 min as the minimum and maximum waiting times during each step change, respectively. For dark respiration, light in the LI-6400XT leaf chamber was switched off for 20 min before measurements were made. There were three or four biological replicates, i.e. plants per species. The initial slope (IS) of each *A*-*C*_i_ curve was estimated by fitting a linear model to the initial 3–4 linear data points such that maximum *C*_i_ was about 55 μl l^−1^ at all leaf temperatures. The maximum CO_2_-saturated rate of each *A*-*C*_i_ curve at each leaf temperature was considered as the CO_2_-saturated rate (CSR).

### Calculation of photosynthetic carbon isotope discrimination, leakiness, and C_4_ cycle rate

Bundle-sheath leakiness was determined by measuring real-time ^13^CO_2_/^12^CO_2_ carbon isotope discrimination using a LI-6400XT attached to a tunable diode laser (TDL, model TGA100, Campbell Scientific, Inc., Logan, Utah, USA) under similar conditions to the steady-state measurements. For estimation of TDL precision, we used the δ^13^C value of the LI-6400XT reference gas for *S. bicolor*. The mean SD of repeated measurements for δ^13^C were 0.24, 0.14, 0.20, and 0.28‰ at 18, 25, 32, and 40 ^o^C, respectively, with an overall SD of 0.21‰. Photosynthetic carbon isotope discrimination (∆) was calculated according to Evans *et al.* (1986):

$$\Delta \;=\frac{\xi \;\cdot (\delta_{o}-\delta_{e})}{1+\delta_{o}-\xi \;\cdot \;\left(\delta_{o}-\delta_{e}\right)}$$ (1)

$$\xi =\;\frac{C_{e}}{C_{e}-C_{o}}$$ (2)

where δ_e_, δ_o_, *C*_e_, and *C*_o_ are the δ^13^C (δ) and CO_2_ mol fraction (*C*) measured with the TDL of the air entering (e) and leaving (o) the leaf chamber. Leakiness (ϕ) was calculated using the model of Farquhar (1983) as modified by Pengelly *et al* (2010) and Pengelly *et al* (2012). The slightly modified equation used here is:

$$\phi =\;\frac{\left(\frac{1-t}{1+t}\right)\;\cdot \Delta \;-\;\frac{a^{\prime }}{1+t\;}\;-\;\left(a_{i}-b_{4}^{\prime }\right)\;\cdot \;\frac{A}{g_{m}\;\cdot \;C_{a}}\;-\;\left(b_{4}^{\prime }-\;\frac{a\prime }{1+t}\right)\;\cdot \;\frac{C_{i}}{\;C_{a}}}{(b_{3}^{\prime }-s)\;\cdot \;\left(\frac{C_{i}}{C_{a}}\;-\;\frac{A}{C_{a}\;\cdot \;g_{m}}\right)}$$ (3)

where Δ is the photosynthetic carbon isotope discrimination measured by the TDL. *a*_i_ is the fractionation factor associated with the dissolution of CO_2_ and its diffusion through water. Here, we assume that *s*=*a*_i_. The term *t*, which represents ternary effects of transpiration rate on the carbon isotope discrimination during CO_2_ assimilation, is defined according to Farquhar and Cernusak (2012) as:

$$t=\;\frac{\left(1+a\prime \right)\;\cdot \;E}{2g_{\text{ac}}^{t}}$$ (4)

where *E* is the transpiration rate and *g*^t^_ac_ is the total conductance to CO_2_ diffusion including boundary layer and stomatal conductance (von Caemmerer and Farquhar, 1981).

The combined fractionation factor through the leaf boundary layer and stomata is denoted by *a*′:

$$a\prime =\frac{a_{b}\;\cdot \;\left(C_{a}-\;C_{\text{ls}}\right)+\;a\;\cdot \;(C_{\text{ls}}-C_{i})}{C_{a}-C_{i}}$$ (5)

where: *C*_a_, *C*_i_, and *C*_ls_ are the ambient, intercellular, and leaf surface CO_2_ partial pressures, respectively; *a*_b_ (2.9‰) is the fractionation occurring through diffusion in the boundary layer; *s* (1.8‰) is the fractionation during leakage of CO_2_ out of the bundle sheath; and *a* (4.4‰) is the fractionation due to diffusion in air (Evans *et al.*, 1986).

$$b_{3}^{\prime }=\;b_{3}-e\;\cdot \left(\frac{R_{d}}{A+R_{d}}-\frac{0.5\;\cdot \;R_{d}}{A+0.5\;\cdot \;R_{d}}\right)-f\cdot \frac{\Gamma *}{C_{s}}$$ (6)

and

$$b_{4}^{\prime }=b_{4}-e\cdot \frac{\;0.5\;\cdot \;R_{d}}{\left(A+0.5\;\cdot \;R_{d}\right)}$$ (7)

where: *b*_3_ is the fractionation by Rubisco (30‰); *b*_4_ is the combined fractionation of the conversion of CO_2_ to HCO_3_^−^ and PEP carboxylation (–5.74‰ at 25 °C); *f* is the fraction associated with photorespiration; Г* is the CO_2_ partial pressure where rate of photorespiratory CO_2_ release balances the rate of carboxylation; and *C*_s_ is the CO_2_ partial pressure in the BSC. The fractionation factor *e* associated with respiration was calculated from the difference between δ^13^C in the CO_2_ cylinder used during experiments (–5.6‰) and that in the atmosphere under growth conditions (–8‰) (Tazoe *et al.*, 2008). *A* and *R*_d_ denote the CO_2_ assimilation rate and daytime respiration, respectively; *R*_d_ was assumed equal to the measured dark respiration (Atkin *et al.*, 1997). We considered mesophyll conductance (*g*_m_) to be 1.78 mol m^−2^ s^−1^ at 30 ^o^C (Barbour *et al.*, 2016; Ubierna *et al.*, 2017). The temperature dependency of *g*_m_ was accounted for by using the Arrhenius function:

$$g_{m}=g_{m25\cdot }e^{E_{a}\cdot \;\frac{T_{k}-298.15}{298.15\;\cdot \;R\;\cdot \;T_{k}}}$$ (8)

where *T*_k_ is the leaf temperature in K; *g*_m25_ is the mesophyll conductance at 25 °C; *E*_a_ is the activation energy, taken as 40.6 kJ mol^−1^ (the value for for *Zea mays*) (Ubierna *et al.*, 2017); and R is the universal gas constant (0.008314 kJ K^−1^). In this study, leaf gas exchange was measured at high light and it was assumed that $f\frac{\Gamma *}{C_{s}}$=0 (Pengelly *et al.*, 2010, 2012; Ubierna *et al.*, 2013; von Caemmerer *et al.*, 2014).

We used ϕ, *A* and *R*_d_ to calculate the C_4_ cycle rate (*V*_p_), assuming that Rubisco oxygenation approximated to zero, i.e. *V*_o_≈0 (Pengelly *et al.*, 2012).

$$V_{p}=\frac{A+0.5R_{d}}{1-\phi }$$ (9)

### Determination of Rubisco and soluble protein contents

Following gas exchange measurements, replicate leaf discs were cut and rapidly frozen in liquid nitrogen and then stored at –80 °C until analysis. Each leaf disc was extracted in 0.8 ml of ice-cold extraction buffer [50 mM EPPS-NaOH (pH 7.8), 5 mM DTT, 5 mM MgCl_2_, 1 mM EDTA, 10 μl protease inhibitor cocktail (Sigma), 1% (w/v) polyvinyl polypyrrolidone] using a 2-ml Tenbroeck glass homogenizer kept on ice. The extract was centrifuged at 15 000 rpm for 1 min and the supernatant was used for enzyme activity (see below), Rubisco content, and soluble protein assays. For Rubisco content, subsamples were activated in buffer [50 mM EPPS (pH 8.0), 10 mM MgCl_2_, 2 mM EDTA, 20 mM NaHCO_3_], and content was estimated by the irreversible binding of [^14^C]-CABP to the fully carbamylated enzyme (Sharwood *et al.*, 2008). Extractable soluble proteins were measured using the Pierce Coomassie Plus (Bradford) protein assay kit (Thermo scientific, Rockford, USA).

### *In vitro* thermal response of Rubisco and PEPC activities

Enzymatic assays were done for Rubisco and PEPC at 18, 25, 34 and 40 °C. To achieve the target temperatures, cuvettes with assay buffer were kept in incubators for 20 min (18 °C), 10 min (25 °C), 10 min (34 °C), and 5 min (40 °C). The maximal *in vitro* activities of Rubisco (*V*_cmax_) and PEPC (*V*_pmax_) were measured spectrophotometrically as described previously (Jenkins *et al.*, 1987; Ashton *et al.*, 1990; Sharwood *et al.*, 2008, 2014, 2016*b*; Pengelly *et al.*, 2010). Briefly, *V*_cmax_ was measured in assay buffer [50 mM EPPS-NaOH (pH 8), 10 mM MgCl_2_, 0.5 mM EDTA, 1 mM ATP, 5 mM phosphocreatine, 20 mM NaHCO_3_, 0.2 mM NADH, 50 U creatine phosphokinase, 0.2 mg carbonic anhydrase, 50 U 3-phosphoglycerate kinase, 40 U glyceraldehyde-3-phosphate dehydrogenase, 113 U triose-phosphate isomerase, 39 U glycerol-3-phosphate dehydrogenase] and the reaction was initiated by the addition of 0.22 mM ribulose-1, 5-bisphosphate (RuBP). *V*_pmax_ was measured in assay buffer [50 mM EPPS-NaOH (pH 8.0), 0.5 mM EDTA, 10 mM MgCl_2_, 0.2 mM NADH, 5 mM glucose-6-phosphate, 0.2 mM NADH, 1 mM NaHCO_3_, 1 U MDH] after the addition of 4 mM PEP. Maximal activities of Rubisco and PEPC were calculated by monitoring the decrease of NADH absorbance at 340 nm using a UV-VIS spectrophotometer (model 8453, Agilent Technologies Australia, Mulgrave, Victoria).

### Temperature dependency

The temperature response of *A*, CSR, IS, *V*_pmax_, and *V*_cmax_ were fitted in the R software (R Development Core Team, 2015) using two different equations, as follows. A modified form of the Arrhenius equation was used to fit the temperature dependence, which yields a peak function (Harley *et al.*, 1992; Crous *et al.*, 2013), and is given by the following equation:

$$f\left(T_{k}\right)=k_{25}\cdot e^{\left[\frac{E_{a}\cdot \left(T_{k}-298\right)}{298\cdot T_{k}\cdot R}\right]}.\left[\frac{1+e^{\left(\frac{298\cdot \Delta S-H_{d}}{298\cdot R}\right)}\;}{1+\;e^{\left(\frac{T_{k}\cdot \Delta S-H_{d}}{T_{k}\cdot R}\right)}}\right]$$ (10)

where, *T*_k_ is the measurement temperature (either leaf or assay buffer) in K; *E*_a_ is the activation energy (kJ mol^−1^) and represents the expansion for the initial part of the temperature response curve; *k*_25_ is the parameter at 25 °C; *H*_d_ is the deactivation energy (kJ mol^−1^); R is the universal gas constant; and *∆S* (kJ mol^−1^ K^−1^) is the entropy term, which describes the peak part of the curve. *H*_d_ and *∆S* together describe the rate of decrease in the function above the optimum. To avoid over-parameterization, the *H*_d_ of all parameters was set as constant (200 kJ mol^−1^) for model fitting, as has been done previously (Medlyn *et al.*, 2002; Crous *et al.*, 2013).

The outputs from the modified Arrhenius and the June *et al.* (2004) equations are compared in Supplementary Fig. S2. The modified form of the Arrhenius equation over-estimates the optimum parameter rate, *P*_opt_, at the temperature optimum, *T*_opt_. Consequently, *T*_opt_ and *P*_opt_ were derived by following the June *et al.* (2004) equation:

$$f\left(T\right)=P_{\text{opt}}\cdot e^{-{\left(\frac{T-T_{\text{opt}}}{\Omega }\right)}^{2}}$$ (11)

where *T* is the measurement temperature (either leaf or assay buffer) of parameter *P* in °C; *P*_opt_ is the optimum parameter rate at the optimum temperature, *T*_opt_; and Ω is the difference in temperature from *T*_opt_ at which the parameter falls to *e*^−1^ (0.37) of its value at *T*_opt_. A smaller value of Ω means a narrower peak. This equation effectively assumes that the reversible processes are symmetrical around the optimum temperature.

### Statistical analyses

The statistical design included species, subtypes, and leaf temperature as factors. Due to the limited number of species used, the statistical analyses could not incorporate phylogenetic or evolutionary effects. Gas exchange measurements and enzyme activity measurements were performed on three or four replicates at each temperature. The coefficients derived by fitting equations (10) and (11) for each parameter were used to test for differences between the thermal responses of species and subtypes. The effect of species was compared using a linear model with type-II ANOVA. The effect of subtype was compared using a linear mixed-effect model using the lme4 package (https://rdrr.io/cran/lme4/) in R (R Development CoreTeam, 2015). Significance tests were performed using type-II ANOVA. Coefficient means were ranked using *post hoc* Tukey tests.

Recent studies have highlighted the flexibility of the decarboxylases; in particular, the expression of PEP-CK activity in NADP-ME subtypes (Wingler *et al.*, 1999; Furbank, 2011; Bellasio and Griffiths, 2014). Except for a relatively high activity in *Z. mays* (one-third of total decarboxylase activity), PEP-CK activity for most NADP-ME subtypes including those used in the current study does not exceed 5–25% of the total decarboxylase activity (see Supplementary Fig. S3). In addition, among the three species in the NADP-ME subtype, two are highly domesticated (*S. bicolor* and *Z. mays*), and *Z. mays* and *Cenchrus ciliaris* had appreciable secondary PEP-CK activity. However, the three NADP-ME species did not separate according to their known PEP-CK activity or domestication for the parameters collected (Supplementary Fig. S3). Consequently, for the purposes of the current study, it was valid to analyse the NADP-ME species as a single group rather than as one made up of multiple subgroups.

## Results

### Leaf gas exchange at 25 °C

**Fig. 1.**
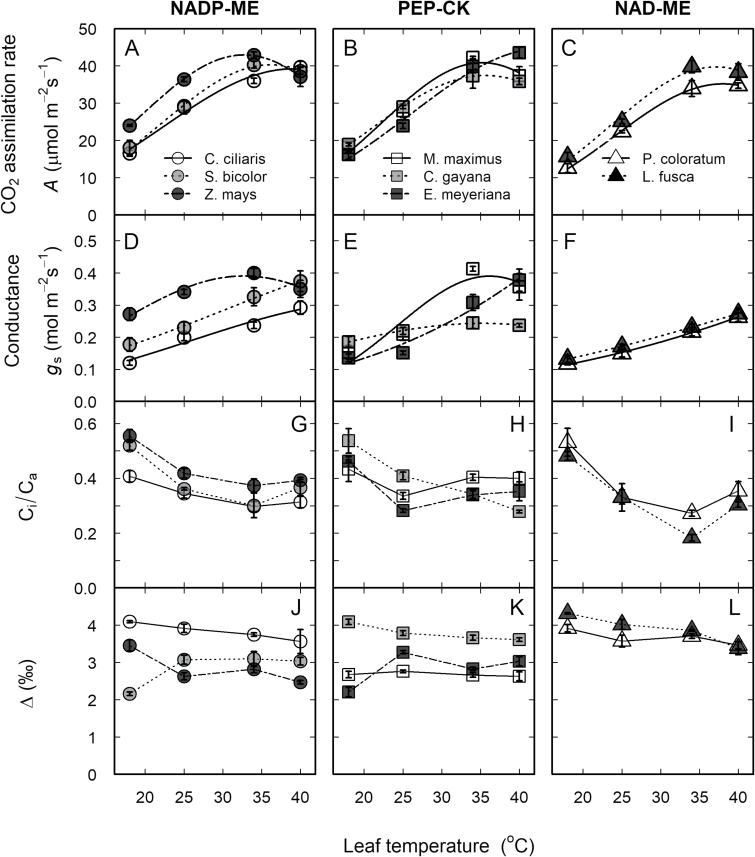
Thermal responses of leaf gas exchange and photosynthetic carbon isotope discrimination in eight C_4_ grasses. (A–C) CO_2_ assimilation rate, *A*, (D–F) stomatal conductance, *g*_*s*_, (G–I) ratio of intercellular to ambient CO_2_, *C*_i_/*C*_a_, and (J–L) photosynthetic carbon isotope discrimination, Δ, as a function of leaf temperature for the C_4_ subtypes NADP-ME, PEP-CK, and NAD-ME (as indicated). The grasses were grown in a common glasshouse. Data in (A–F) were fitted according to June *et al.* (2004) and the derived constants are shown in Table 2. Leaves were measured at 1800 μmol m^–2^ s^–1^ PPFD and 400 μl l^–1^ CO_2._ Values are means of 3–4 replicates ±SE.

Leaf gas exchange parameters were measured at ambient CO_2_ and near-saturating light intensity for all the C_4_ grasses concurrently with stable carbon isotope discrimination. At 25 °C, net CO_2_ assimilation rate (*A*), stomatal conductance (*g*_s_), the ratio of internal to atmospheric CO_2_ partial pressure (*C*_i_/*C*_a_), dark respiration (*R*_d_), and photosynthetic carbon isotope discrimination (∆) all varied significantly among the species (*P*<0.05; Fig. 1, Supplementary Table S1). Only *A* varied significantly (*P*<0.05) according to the C_4_ subtype, with NAD-ME species having lower *A* relative to NADP-ME and PEP-CK species. Photosynthetic carbon isotope discrimination was lowest in *Megathyrsus maximus* and *Z. mays*, and highest in *C. ciliaris*, *Chloris gayana*, and *Leptochloa fusca*.

### Temperature response of leaf gas exchange

**Fig. 2.**
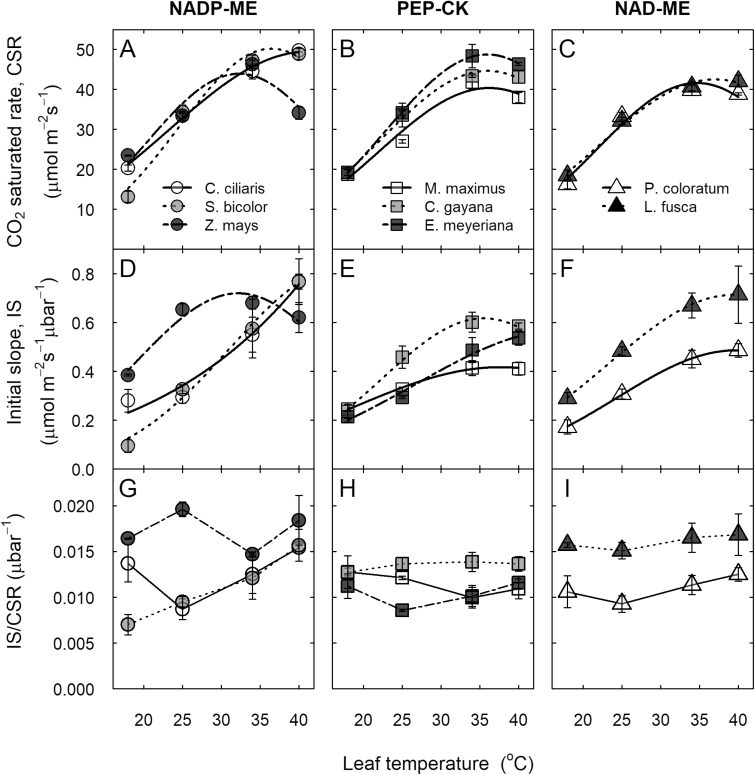
Thermal responses of the CO_2_-saturated rate (CSR) and the initial slope of the *A*-*C*_i_ curve (IS) in eight C_4_ grasses. (A–C) CO_2_-saturated rate, CSR, (D–F) initial slope of the CO_2_ response curve, IS, and (G–I) the IS/CSR ratio as a function of leaf temperature for the C_4_ subtypes NADP-ME, PEP-CK, and NAD-ME (as indicated). Data in (A–F) were fitted according to June *et al.* (2004) and the derived parameters are shown in Table 2. Leaves were measured at 1800 μmol m^–2^ s^–1^ PPFD. Values are means of 3–4 replicates ±SE

The initial slope (IS) and CO_2_-saturated rate (CSR) of photosynthetic response to intercellular CO_2_ partial pressure (from *A*-*C*_i_ curves) were estimated at four temperatures (see Supplementary Fig. S4). At 25 °C, IS, CSR, and IS/CSR varied significantly with species but not with subtypes. *Zea mays* and *L. fusca* had the highest IS and IS/CSR, while *M. maximus* had the lowest CSR relative to the other C_4_ species (Fig. 2, Supplementary Table S2).

### Rubisco and PEPC measurements at 25 °C

**Fig. 3.**
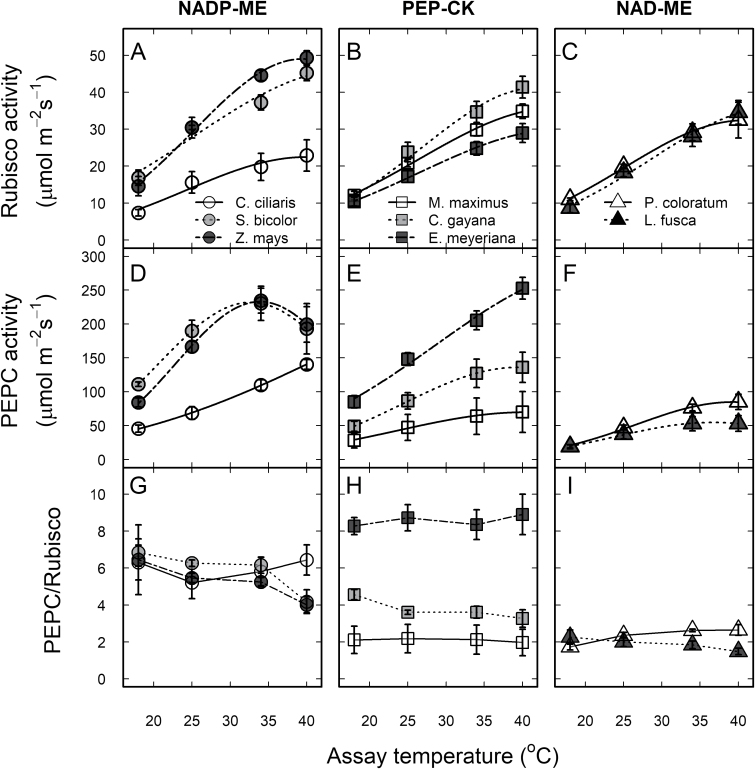
Thermal responses of photosynthetic enzyme activities in eight C_4_ grasses. (A–C) Rubisco activity, (D–F) PEPC activity, and (G–I) the PEPC/Rubisco activity ratio as a function of temperature for the C_4_ subtypes NADP-ME, PEP-CK, and NAD-ME (as indicated). For each extract, the temperature responses of both enzymes were measured at 18, 25, 34, and 40 °C. Data in (A–F) are fitted according to June *et al.* (2004) and the derived parameters are shown in Table 2. Values are means of 3–4 replicates ±SE.

Maximal activities of PEPC (*V*_pmax_) and Rubisco (*V*_cmax_) were measured on the same leaves used for gas exchange. At 25 °C, *V*_cmax_ and *V*_pmax_ were highest in the two NADP-ME subtypes *S. bicolor* and *Z. mays*. *V*_pmax_ and *V*_pmax_/*V*_cmax_ were lowest in the two NAD-ME subtypes along with *M. maximus* (PEP-CK) relative to the other species (Fig. 3, Supplementary Table S2). In line with earlier studies, we obtained an average extraction yield of 80% for *V*_cmax_ (maximal CO_2_ assimilation/Rubisco activity) for wild C_4_ grasses (Meinzer and Zhu, 1998; Kingston-Smith *et al.*, 1999; Crafts-Brandner and Salvucci, 2002; Dwyer *et al.*, 2007).

Leaf Rubisco content varied among the species (3.8–7.8 µmol sites m^–2^), constituting about 6% of the soluble protein fraction (Supplementary Table S3). Among all the species, Rubisco and soluble protein contents were highest in *Z. mays* and *Panicum coloratum* and lowest in *C. ciliaris*, *Eriochloa meyeriana*, and *L. fusca*. Rubisco activation measured at 25 °C tended to be higher in NADP-ME (69%) compared to PEP-CK (53%) and NAD-ME (54%) subtypes.

### Thermal responses of photosynthetic parameters

The short-term thermal responses of *A*, IS, CSR, *V*_pmax_, and *V*_cmax_ were well characterized by the modified Arrhenius and June *et al.* (2004) equations (equations 10 and 11, Supplementary Figs S2 and S5–S8). No subtype effect was observed for the activation energy (*E*_a_), entropy factor (*∆S*), parameter at 25 °C (*k*_25_), the optimum parameter (*P*_opt_) at the optimum temperature (*T*_opt_), or the width of curvature around *T*_opt_ (Ω) derived for IS, CSR, *V*_pmax_, and *V*_cmax_ (Table 2).

**Table 2.** Summary of thermal responses of photosynthetic parameters for eight C_4_ grasses.Coefficients are derived by fitting equation (10) (modified Arrhenius) and equation (11) (June *et al.*, 2004). *E*_a_ is the activation energy (kJ mol^−1^), ∆S is an entropy term (kJ mol^−1^), *k*_25_ and *P*_opt_ are the parameter values at 25 °C and its optimum temperature (*T*_opt_), respectively. Ω (°C) is the difference in temperature from *T*_opt_ at which the parameter falls to *e*^−1^ (0.37) of its value at *T*_*opt*_. Values are means of three replicates ±SE. The ranking (from lowest =a) of species/subtypes within each individual row was derived using a multiple-comparison Tukey’s *post hoc* test. Values followed by the same letter are not significantly different at the 5% level. *P*-values show significance levels derived by fitting a linear model for all the C_4_ species and a linear mixed-effect model for the three C_4_ subtypes for each parameter: ns, not significant (*P*>0.05); * *P*<0.05; ** *P*<0.01; *** *P*<0.001.

Parameter	Const.	NADP-ME			PEP-CK			NAD-ME		Subtype			*P*-value	
		*C. ciliaris*	*S. bicolor*	*Z. mays*	*M.maximus*	*Ch.gayana*	*E.meyeriana*	*P.coloratum*	*L. fusca*	NADP-ME	PEP-CK	NAD-ME	Species	Subtype
CO_2_ assimilation rate, *A* (µmol m^–2^ s^-1^)	*E*_a_	36 ± 1a	42 ± 7a	34 ± 3a	50 ± 7a	36 ± 7a	48 ± 7a	51 ± 4a	54 ± 13a	37 ± 3a	45 ± 4ab	52 ± 5b	ns	*
	∆*S*	0.63 ± 0a	0.63 ± 0a	0.64 ± 0a	0.64 ± 0a	0.63 ± 0.01a	0.63 ± 0a	0.63 ± 0a	0.64 ± 0.01a	0.63 ± 0a	0.63 ± 0a	0.64 ± 0a	ns	ns
	*k*_25_	26 ± 1b	28 ± 1b	34 ± 1c	28 ± 1b	28 ± 1b	24 ± 1ab	21 ± 1a	25 ± 0ab	29 ± 1a	27 ± 1a	23 ± 1a	***	0.1
	*P*_opt_	40 ± 1abc	42 ± 0bc	43 ± 1bc	41 ± 1abc	38 ± 1ab	46 ± 3c	36 ± 1a	41 ± 0abc	42 ± 1a	41 ± 1a	38 ± 1a	**	ns
	*T*_opt_	41 ± 3a	39 ± 4a	33 ± 1a	35 ± 1a	37 ± 3a	43 ± 6a	39 ± 2a	38 ± 3a	38 ± 2a	38 ± 2a	38 ± 1a	ns	ns
	Ω	25 ± 3a	24 ± 6a	20 ± 2a	18 ± 2a	23 ± 4a	22 ± 5a	20 ± 2a	19 ± 4a	23 ± 2a	21 ± 2a	20 ± 2a	ns	ns
CO_2_ saturated rate, CSR (µmol m^–2^ s^–1^)	*E*_a_	36 ± 1a	58 ± 2b	45 ± 1ab	45 ± 7ab	42 ± 5ab	49 ± 7ab	44 ± 2ab	37 ± 0ab	47 ± 3a	45 ± 3a	41 ± 2a	*	ns
	∆*S*	0.63 ± 0a	0.64 ± 0b	0.64 ± 0b	0.64 ± 0b	0.63 ± 0ab	0.64 ± 0b	0.64 ± 0b	0.63 ± 0ab	0.64 ± 0a	0.64 ± 0a	0.64 ± 0a	**	ns
	*k*_25_	31 ± 1ab	29 ± 1a	34 ± 0b	28 ± 0a	31 ± 1ab	32 ± 1ab	29 ± 1a	30 ± 1ab	32 ± 1a	30 ± 1a	29 ± 1a	ns	ns
	*P*_opt_	51 ± 1c	50 ± 1bc	44 ± 1abc	40 ± 2a	45 ± 1ac	49 ± 2bc	42 ± 1a	43 ± 2ab	48 ± 1a	45 ± 1a	42 ± 1a	***	ns
	*T*_opt_	42 ± 3b	36 ± 0ab	32 ± 0a	36 ± 0ab	36 ± 1ab	36 ± 1ab	35 ± 0a	36 ± 1ab	37 ± 2a	36 ± 0a	35 ± 0a	**	ns
	Ω	26 ± 3c	17 ± 0bc	17 ± 0ac	21 ± 2a	20 ± 2ac	19 ± 2bc	18 ± 0a	21 ± 1ab	20 ± 2a	20 ± 1a	19 ± 1a	*	ns
Rubisco activity, *V*_cmax_ (umol m^–2^ s^–1^)	*E*_a_	42 ± 3ab	36 ± 6a	52 ± 6ab	38 ± 1ab	52 ± 4ab	42 ± 5ab	47 ± 3ab	58 ± 4b	43 ± 3a	44 ± 3a	52 ± 3a	*	ns
	∆S	0.63 ± 0a	0.62 ± 0a	0.63 ± 0a	0.63 ± 0a	0.63 ± 0a	0.63 ± 0a	0.63 ± 0a	0.63 ± 0a	0.63 ± 0a	0.63 ± 0a	0.63 ± 0a	ns	ns
	*k*_25_	13 ± 2a	27 ± 3b	27 ± 1b	19 ± 1ab	21 ± 2ab	16 ± 1a	19 ± 1ab	17 ± 1a	23 ± 3a	19 ± 1a	18 ± 1a	***	ns
	*P*_opt_	24 ± 4a	47 ± 1b	49 ± 2b	37 ± 3ab	44 ± 3ab	30 ± 3ab	35 ± 6ab	39 ± 7ab	40 ± 4a	37 ± 3a	37 ± 4a	**	ns
	*T*_opt_	42 ± 4a	45 ± 2a	39 ± 0a	47 ± 3a	44 ± 4a	46 ± 1a	42 ± 4a	44 ± 4a	42 ± 2a	46 ± 2a	43 ± 3a	ns	ns
	Ω	23 ± 3a	28 ± 2b	20 ± 1b	29 ± 2ab	23 ± 3ab	27 ± 2ab	22 ± 2ab	22 ± 3ab	24 ± 2a	26 ± 1a	22 ± 2a	ns	ns
Initial slope, IS (µmol m^–2^ s^–1^ µbar^–1^)	*E*_a_	47 ± 2ab	70 ± 9b	34 ± 13a	27 ± 3a	48 ± 5ab	43 ± 7ab	47 ± 3ab	52 ± 8ab	53 ± 8a	39 ± 4a	49 ± 3a	**	ns
	∆*S*	0.62 ± 0.01a	0.63 ± 0.01a	0.63 ± 0.01a	0.63 ± 0a	0.64 ± 0a	0.63 ± 0.01a	0.63 ± 0a	0.64 ± 0a	0.63 ± 0a	0.63 ± 0a	0.64 ± 0a	ns	0.06
	*k*_25_	0.35 ± 0.05ac	0.27 ± 0.04a	0.57 ± 0.02d	0.32 ± 0.02ac	0.41 ± 0.04bc	0.3 ± 0.02ab	0.29 ± 0.01ab	0.47 ± 0.01cd	0.38 ± 0.05a	0.35 ± 0.02a	0.36 ± 0.04a	***	ns
	*P*_opt_	1.67 ± 0.68b	0.9 ± 0.1ab	0.73 ± 0.01a	0.42 ± 0.03a	0.63 ± 0.03a	0.66 ± 0.11a	0.49 ± 0.04a	0.7 ± 0.08ab	1.03 ± 0.2a	0.58 ± 0.06a	0.57 ± 0.05a	*	ns
	*T*_opt_	72 ± 14b	48 ± 5ab	33 ± 2a	38 ± 2a	36 ± 2a	51 ± 8ab	40 ± 2a	34 ± 2a	49 ± 7a	38 ± 2a	42 ± 3a	**	ns
	Ω	39 ± 4b	22 ± 3ab	20 ± 3a	28 ± 4ab	19 ± 2a	31 ± 5ab	22 ± 2ab	17 ± 0a	25 ± 3a	20 ± 2a	26 ± 3a	*	ns
PEPC activity, *V*_pmax_ (µmol m^–2^ s^–1^)	*E*_a_	39 ± 1a	44 ± 3a	61 ± 6bc	39 ± 5a	47 ± 2ab	39 ± 1a	65 ± 3c	51 ± 2ac	48 ± 4a	42 ± 2a	58 ± 4a	***	ns
	∆*S*	0.62 ± 0a	0.64 ± 0cd	0.64 ± 0d	0.63 ± 0bc	0.63 ± 0bd	0.62 ± 0ab	0.64 ± 0cd	0.64 ± 0 cd	0.63 ± 0a	0.63 ± 0a	0.64 ± 0a	***	ns
	*k*_25_	71 ± 6a	186 ± 6c	156 ± 7bc	44 ± 18a	81 ± 12a	135 ± 11b	42 ± 3a	37 ± 7a	138 ± 17a	87 ± 15a	40 ± 4a	***	0.07
	*P*_opt_	197 ± 19bcd	255 ± 23d	234 ± 18cd	70 ± 30a	138 ± 23ac	288 ± 33d	90 ± 10ab	61 ± 14a	229 ± 13a	165 ± 35a	76 ± 10a	***	0.07
	*T*_opt_	61 ± 5c	33 ± 1a	34 ± 1a	40 ± 1ab	40 ± 2ab	51 ± 3bc	40 ± 3ab	37 ± 1a	43 ± 5a	43 ± 2a	39 ± 2a	***	ns
	Ω	36 ± 2bcd	17 ± 0d	16 ± 1cd	24 ± 3a	21 ± 1ac	30 ± 2d	18 ± 2ab	19 ± 0a	23 ± 3a	25 ± 2a	19 ± 1a	***	ns

For CO_2_ assimilation rate, the range of variation observed for *T*_opt_ (33–43 °C), *E*_a_ (34–54 kJ mol^−1^), and Ω (18–24 °C) was not significantly different among all the species (Fig. 1A–C, Table 2). *E*_a_ was significantly lower (*P*<0.05) in NADP-ME subtypes (37 kJ mol^−1^) relative to NAD-ME (52 kJ mol^−1^). Conversely, *k*_25_ tended to higher (*P*=0.1) in NADP-ME subtypes relative to NAD-ME. For CSR, *C. ciliaris* had the highest *P*_opt_, *T*_opt_, and Ω, while *P. coloratum* and *M. maximus* had the lowest *P*_opt_, and *Z. mays* and *P. coloratum* had the lowest *T*_opt_. For IS, *C. ciliaris* had the highest *T*_opt_, *P*_opt_, and Ω, while *E*_a_ was highest in *S. bicolor* and lowest in *Z. mays* and *M. maximus* relative to the other species (Fig. 2, Table 2). Overall, the ratio IS/CSR was not affected by leaf temperature, except in *S. bicolor* where IS/CSR increased with temperature (Fig. 2, Supplementary Table S2).

For the temperature response of *in vitro V*_cmax_, no parameter varied significantly according to the biochemical subtype, while *E*_a_, *k*_25_, and *P*_opt_ varied among all the species (Table 2). Rubisco *E*_a_ was highest in *L. fusca* (58 kJ mol^−1^) and lowest in *S. bicolor* (36 kJ mol^−1^), while Rubisco *k*_25_ and *P*_opt_ were highest in *S. bicolor* and *Z. mays* and lowest in *C. ciliaris* (Fig. 3A–C, Table 2).

All parameters describing the thermal response of *in vitro V*_pmax_ varied significantly among all the species. In addition, *P*_opt_ and *k*_25_ tended to be lowest (*P*<0.07) in NAD-ME, intermediate in PEP-CK, and highest in NADP-ME subtypes (Fig. 3D–F, Table 2). Overall, the ratio IS/CSR was unaffected by leaf temperature (Fig. 3, Supplementary Table S2).

### Photosynthetic carbon isotope discrimination and bundle sheath leakiness

**Fig. 4.**
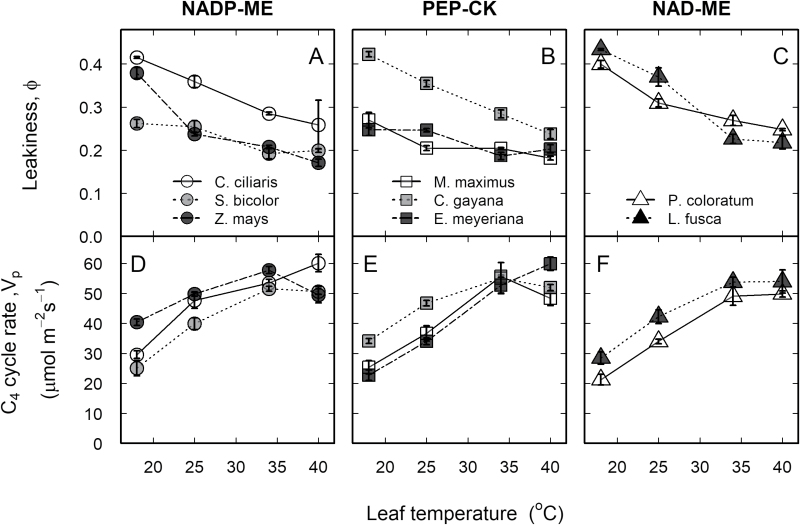
Thermal responses of bundle sheath leakiness and the C_4_ cycle rate in eight C_4_ grasses. (A–C) Estimated leakiness (ϕ) and (D–F) C_4_ cycle rate (*V*_p_) as a function of leaf temperature for the C_4_ subtypes NADP-ME, PEP-CK, and NAD-ME (as indicated). The grasses were grown in a common glasshouse. Leaves were measured at 1800 μmol m^–2^ s^–1^ PPFD and 400 μl l^–1^ CO_2_. Values are means of 3–4 replicates ±SE.

Photosynthetic carbon isotope discrimination (∆) was unchanged between 25 and 40 °C and increased significantly at 18 °C for most of the species, except in *S. bicolor* and *E. meyeriana* where it decreased at 18 °C (Fig. 1J–LSupplementary Table S1). Overall, the NAD-ME subtypes showed higher Δ compared to NADP-ME and PEP-CK. Leakiness (ϕ) was higher at the two lowest temperatures (18 and 25 °C) relative to the two highest temperatures (34 and 40 °C), with ϕ at 40 °C being similar among all the species (Fig. 4A–C, Supplementary Table S1). *Sorghum bicolor*, *M. maximus*, and *E. meyeriana* had lower ϕ relative to the other species at both 18 and 34 °C. Generally, most leakiness values ranged between 35% at 18 °C and 10% at 40 °C (Supplementary Fig. S11). The estimated C_4_ cycle rate (*V*_p_) increased between 18 and 35 °C and was similar between 35 and 40 °C for all the species (Fig. 4D–F, Supplementary Table S1).

### Relationships among photosynthetic parameters

*In vivo* estimates of CO_2_ assimilation rate (*A*), stomatal conductance (*g*_s_), CO_2_-saturated rate (CSR), initial slope (IS), and leakiness (ϕ), and *in vitro* measurements of *V*_cmax_ and *V*_pmax_ were used to assess how *in vivo* and *in vitro* photosynthetic parameters correlated with short-term changes in temperature.

**Fig. 5.**
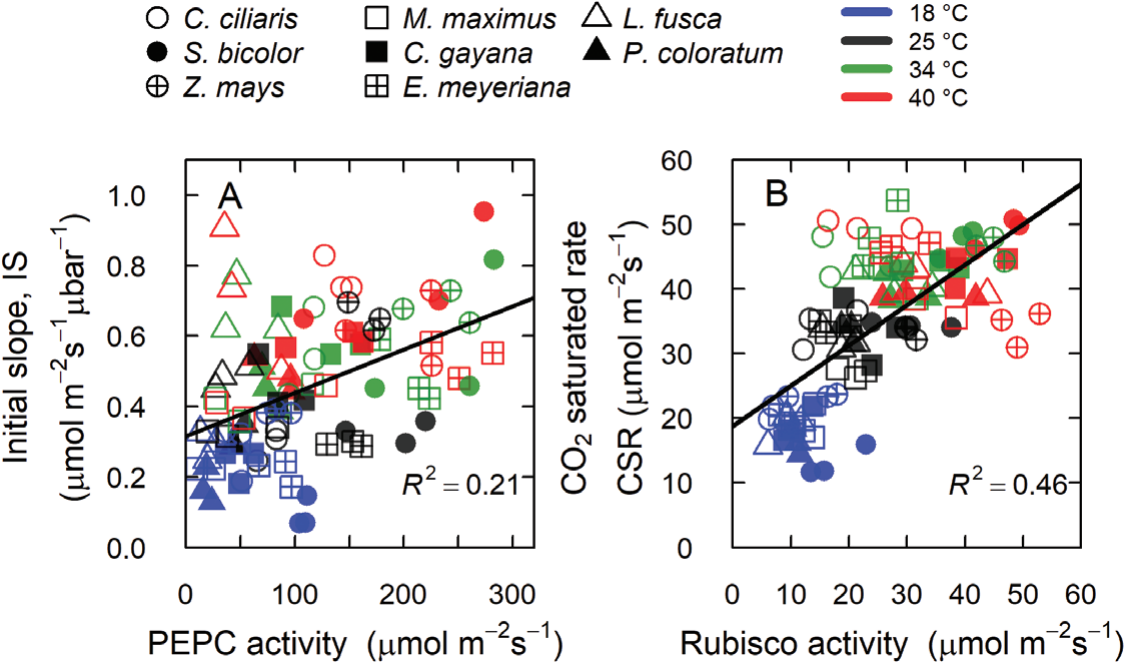
Relationships between measured *in vitro* and *in vivo* estimates of photosynthetic carboxylases. (A) The initial slope of the CO_2_ response curve, IS, versus PEPC activity, and (B) CO_2_ saturated rate, CSR, versus Rubisco activity, measured at 18, 25, 34, and 40 °C (as indicated) in the C_4_ subtypes NADP-ME (circles), PEP-CK (squares), and NAD-ME (triangles). The solid lines represent regressions of all data points.

**Fig. 6.**
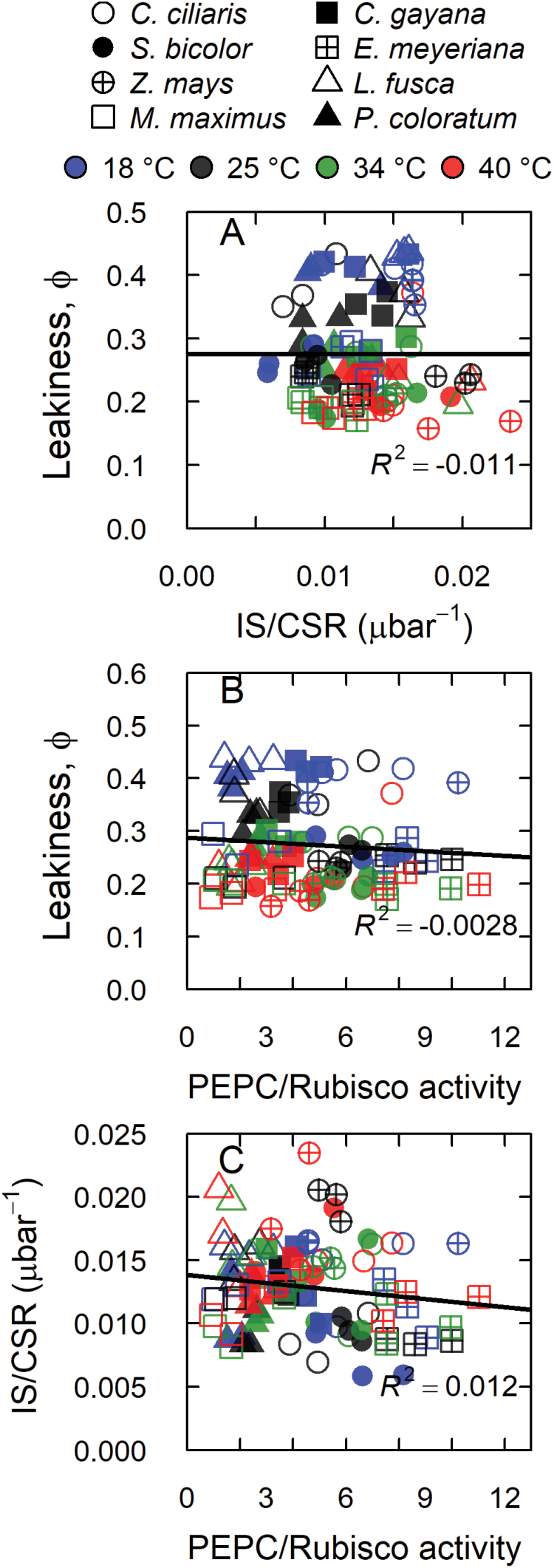
Relationships between leakiness and the activity ratio of *in vitro* or *in vivo* C_4_ and C_3_ cycle carboxylases. (A) Leakiness (ϕ) versus the ratio of the initial slope of the CO_2_ response curve (IS) and the CO_2_-saturated rate (CSR), (B) ϕ versus the ratio of PEPC and Rubisco activities, and (C) the IS/CSR ratio versus the ratio of PEPC and Rubisco activities, measured at 18, 25, 34, and 40 °C (as indicated) in the C_4_ subtypes NADP-ME (circles), PEP-CK (squares), and NAD-ME (triangles). The solid lines represent linear regressions of all data points.

Weak relationships were observed between IS and *V*_pmax_ (*r*^2^=0.21, Fig. 5A) and between CSR and *V*_cmax_ (*r*^*2*^=0.48, Fig. 5B), and a strong relationship was observed between *A* and *g*_s_ (*r*^*2*^=0.78, Supplementary Fig. S10). Leakiness (ϕ) was neither correlated to *V*_pmax_/*V*_cmax_ nor to IS/CSR (Fig. 6A, B). The ratios of IS/CSR and *V*_pmax_/*V*_cmax_ were also not correlated (Fig. 6C).

To shed further light on what controls IS in diverse C_4_ grasses, we used the simplified expression linking *V*_pmax_ with IS derived by Pfeffer and Peisker (1998):

$$\text{PEPC activity}=\frac{K_{p}\cdot \;\text{IS}\cdot g_{m}}{g_{m}-\text{IS}}$$ (12)

where *K*_p_ is the Michaelis–Menten constant for CO_2_. A weak fit was observed between measured *V*_pmax_ values and model predictions when published *K*_p_ values from *Z. mays* were used (Supplementary Fig. S12A, dotted and dashed lines). When equation (12) was solved to simultaneously predict *g*_m_ and *K*_p_ using measured *V*_pmax_ and IS values for *Z. mays* at all four temperatures together with published temperature dependencies of *g*_m_ and *K*_p_ (Bauwe, 1986; Boyd *et al.*, 2015; Ubierna *et al.*, 2017), the observed fit between the measured and modelled data was much improved (Supplementary Fig. S12A, continuous line, and S12B). Fitted values for *Z. mays* at 25 °C for *g*_m_ and *K*_p_ were then found to be 1.1 mol m^−2^ s^−1^ and 115 µbar, respectively (Fig. S12A).

## Discussion

This study analysed the thermal photosynthetic responses of eight diverse C_4_ grasses, deriving constants for thermal dependency that can be incorporated in the C_4_ photosynthesis model (von Caemmerer, 2000). The main aim of the study was to determine whether the C_4_ biochemical subtype influenced the thermal responses of *in vitro* (*V*_pmax_ and *V*_cmax_) and *in vivo* (IS, CSR, and ϕ) photosynthetic parameters that influence the efficiency of the C_4_ CO_2_-concentrating mechanism (CCM). The study also explored whether the ratios of IS/CSR and *V*_pmax_/*V*_cmax_ were correlated with each other or with leakiness across a range of C_4_ species. The answers to these questions were largely negative, as discussed below.

### Thermal photosynthetic responses varied among the C_4_ grasses independently of the biochemical subtype

This study revealed large interspecific variations for most parameters derived from the photosynthetic temperature responses; however, these variations were largely independent of the C_4_ subtype, disagreeing with our prediction (Table 2). There was one exception to this generalization, with the activation energy (*E*_a_) of CO_2_ assimilation being lowest in the NADP-ME subtype. In addition, *V*_pmax_ at 25 °C (*k*_25_) and at *T*_opt_ (*P*_opt_) were marginally (*P*=0.07) lowest in NAD-ME and highest in NADP-ME subtypes. The latter observation is consistent with our previous reports of NAD-ME subtypes generally having lower *V*_pmax_ and *V*_pmax_/*V*_cmax_ (Pinto *et al.*, 2016).

The *E*_a_ for *in vitro V*_cmax_ (36–58 kJ mol^−1^) and *V*_pmax_ (39–65 kJ mol^−1^) and their corresponding *in vivo* CSR (36–58 kJ mol^−1^) and IS (27–70 kJ mol^−1^) varied by 2-fold among the species examined here. Although these values are in line with published estimates for C_3_ (*V*_cmax_) and C_4_ (*V*_cmax_ and *V*_pmax_) species, they tended to lie at the lower end of the spectrum reported for most of the C_4_ grasses used in our study (Ishii *et al.*, 1977; Bernacchi *et al.*, 2001; Medlyn *et al.*, 2002; Sage, 2002; Galmés *et al.*, 2005, 2015; Massad *et al.*, 2007; Walker *et al.*, 2013; Perdomo *et al.*, 2015; Sharwood *et al.*, 2016*a*). For example, higher *E*_*a*_ was reported for *V*_cmax_ (78 kJ mol^−1^) and *V*_pmax_ (95 kJ mol^−1^) activities in the NADP-ME grass *Setaria viridis* (Boyd *et al.*, 2015), while relatively higher values of *E*_a_ were reported for the *in vivo V*_pmax_ of the C_4_ species *Z. mays* and *Andropogon gerardii* (60–77.9 kJ mol^−1^) (Wu and Wedding, 1987; Chen *et al.*, 1994). Interestingly, *E*_a_ was higher for *V*_cmax_ than *V*_pmax_ in *C. ciliaris*, *Ch. gayana*, *E. meyeriana*, and *L. fusca*, while the opposite was observed in *S. bicolor*, *Z. mays*, *M. maximus*, and *P. coloratum*. Similarly, lower *E*_a_ for *V*_cmax_ than *V*_pmax_ was reported in *S. viridis* (*in vitro*) (Boyd *et al.*, 2015), and the opposite trend was reported in *A. gerardii* (*in vivo*) (Chen *et al.*, 1994).

We predicted that thermal photosynthetic responses including leakiness may depend on the biochemical subtypes, based on known differences in Rubisco catalytic properties, PSII activity in the BSCs, suberization of the BSC walls, and possibly other anatomical and biochemical traits. In this study, similar thermal responses for leakiness were observed between the C_4_ subtypes, and ϕ at 40 °C was similar for all the species. Comparable results were obtained in an earlier study using a smaller set of C_4_ grasses measured at a common temperature (Cousins *et al.*, 2008). These results suggest that CCM efficiency is similar among the C_4_ subtypes despite their biochemical and anatomical differences. This was evident despite the potential for increased oxygenation in NAD-ME and PEP-CK species with increases in temperature (Siebke *et al.*, 2003) as a result of significant PSII activity in the BSCs of these two subtypes (Edwards *et al.*, 1976; Ghannoum *et al.*, 2005). It has also been suggested that the absence of suberin in the BSC walls of NAD-ME subtypes is counterbalanced by the greater cytosolic barrier that exists for CO_2_ diffusion through the centripetal arrangement of BSC chloroplasts surrounded by mitochondria towards the vascular bundle side (von Caemmerer and Furbank, 2003). However, little is known about the thermal dependence of the CO_2_ diffusion path from the BSCs to MCs in the different C_4_ subtypes.

In the current study, leakiness tended to be higher at the two lowest (18 and 25 °C) relative to the two highest (34 and 40 °C) temperatures for four out of the eight C_4_ grasses (see Supplementary Fig. S11). Similar results were previously reported for a C_4_ monocot and a C_4_ dicot species (Henderson *et al.*, 1992). To gain further insights about the factors leading to higher leakiness at lower temperatures, we calculated the C_4_ cycle rate (*V*_p_). For all the species, the calculated *V*_p_ increased with temperature, which indicates that increased leakiness at lower temperatures in some of the species is not related to increased pumping of CO_2_ into the BSCs, but is probably due to increased Rubisco limitation. In agreement with this, Sage (2002) showed that C_4_ plants are limited by *V*_cmax_ at low temperature. Taken together, these findings indicate a greater Rubisco limitation at low temperature, which may lead to a greater proportion of CO_2_ leakage out of the BSCs in some C_4_ species (Kubien *et al.*, 2003; Kubien and Sage, 2004), in a manner not explained by the biochemical subtype.

### Maximal *in vitro* activities of PEPC and Rubisco, the initial slope and maximal rate of the *A*-*C*_i_ curves, and leakiness are not correlated across a range of C_4_ grasses and leaf temperatures

Major progress has been made in our understanding of C_3_ photosynthesis following the development of a fully mechanistic model (Farquhar *et al.*, 1980) that was subsequently validated by combining leaf gas exchange measurements with estimates of Rubisco activity and mesophyll conductance (*g*_m_) in wild-type (von Caemmerer and Farquhar, 1981; von Caemmerer *et al.*, 1994; Bernacchi *et al.*, 2002; von Caemmerer and Evans, 2015) and genetically altered plants (Hudson *et al.*, 1992). For C_4_ photosynthesis, model validation has been undertaken mostly under standard conditions using a genetically altered C_4_ dicot, *Flaveria bidentis* (von Caemmerer *et al.*, 1997; Kubien *et al.*, 2003; Pengelly *et al.*, 2012), or under a range of conditions using a limited number of model C_4_ grass species, such as *S. bicolor* (Henderson *et al.*, 1992), *Z. mays* (Yin *et al.*, 2016) and *S. viridis* (Boyd *et al.*, 2015). This study presented an ideal opportunity to undertake model validation using *in vitro* enzyme assays and leaf gas exchange measurements for a range of C_4_ species.

A weak correlation was observed between CSR and *V*_cmax_, while none was found between IS and *V*_pmax_ (Fig. 5), or between *E*_a_ of *V*_cmax_ (or *V*_pmax_) and *E*_a_ of CSR (or IS) across the various C_4_ grasses. This discrepancy is not surprising and reflects the multitude of factors controlling the *A*-*C*_i_ curves besides the maximal *in vitro* activity of Rubisco and PEPC. Other than Rubisco activity and level of activation, CSR is also dependent on RuBP and PEP regeneration, and both limitations are generally indistinguishable at high light (von Caemmerer, 2000). The IS is determined by the maximal PEPC activity (*V*_pmax_), its Michaelis–Menten constant for CO_2_ (*K*_p_), and the mesophyll CO_2_ partial pressure (*C*_m_), which depends on mesophyll conductance (*g*_m_) (von Caemmerer, 2000). The parameters *g*_m_ and *K*_p_ were not measured in this study, but using published values at 25 °C together with their temperature dependencies for *Z. mays* (Bauwe, 1986; Boyd *et al.*, 2015; Ubierna *et al.*, 2017) to model the relationship between IS and *V*_pmax_, a weak fit was observed between measured and predicted *V*_pmax_ values (Supplementary Fig. S12A, dotted and dashed lines). In contrast, when model predictions were made using simultaneously solved *g*_m_ and *K*_p_ values using measured *V*_pmax_ and IS for *Z. mays* at all four temperatures, the observed fit between measured and modelled *V*_pmax_ was much improved (Supplementary Fig. S12A, continuous line, and S12B). This exercise indicated that *g*_m_ and *K*_p_ as well as their temperature responses may vary with growth environment and species (or genotype), as has recently been shown for Rubisco kinetics among these C_4_ grasses (Sharwood *et al.*, 2016*a*). The variation in *g*_m_ and its temperature response in C_3_ species is well established (von Caemmerer and Evans, 2015), and discrepancies for *g*_m_ in C_4_*Z. mays* have been reported (Barbour *et al.*, 2016; Ubierna *et al.*, 2017).

In C_3_ species, IS (Rubisco-limited photosynthesis) is largely insensitive to temperature because both the *K*_m_ and *V*_cmax_ of Rubisco have a Q_10_ of 2 (Berry and Raison, 1981; Sage and Sharkey, 1987). In contrast, we observed a strong temperature response of IS (PEPC-limited) for the C_4_ species examined, indicating that the *E*_a_ for *V*_pmax_ is greater than the *E*_a_ of *K*_p_ (Table 2, Fig. 2D–F). A similar trend has been shown in a recent study (Boyd *et al.*, 2015). In addition, variation among C_4_ species for the *E*_a_ of IS suggests that the ratio between *E*_a_ for *V*_pmax_ and *K*_p_ also differs. This has been partly supported by our data in the case of the *E*_a_ for *V*_pmax_ (Table 2, Fig. 3D–F). These findings indicate that there is a significant diversity for PEPC kinetics among C_4_ species, and warrants further investigation.

The improved relationship between measured and modelled *V*_pmax_ relative to that observed between IS and measured *V*_pmax_ (Supplementary Fig. S12A versus S12B) demonstrates that the C_4_ photosynthesis model, when well parameterized, can accurately predict CO_2_ assimilation rates at limiting *C*_i_ in C_4_ leaves. Given the rapidly improving methodologies for measuring the exchange of ^13^C and ^18^O stable isotopes that allow us to estimate both *g*_m_ and *K*_p_ (von Caemmerer *et al.*, 2004; Boyd *et al.*, 2015; Barbour *et al.*, 2016; Ubierna *et al.*, 2017), the C_4_ photosynthesis model will form a valuable tool for interpreting leaf gas exchange data for any C_4_ species under a wide range of environmental conditions, similarly to what has been widely achieved for C_3_ plants. Further parameterization and validation of the C_4_ photosynthesis model for diverse C_4_ species remains a focus of our current research.

Bundle sheath leakiness (ϕ) depends on *g*_bs_ and the (*C*_bs_–*C*_m_) gradient, which in turn depends on the balance between the activities of PEPC and Rubisco (Henderson *et al.*, 1992; von Caemmerer, 2000). In accordance with model predictions, earlier studies reported a relationship between leakiness and *in vitro V*_pmax_/*V*_cmax_ (Ranjith *et al.*, 1995; Saliendra *et al.*, 1996; Meinzer and Zhu, 1998) and *in vivo* IS/CSR (Gong *et al.*, 2017). These studies used a single species (sugarcane or *Cleistogenes squarrosa*) exposed to soil or atmospheric water deficit, and estimated ϕ from the C-isotope composition of leaf dry matter rather than photosynthetic C-isotope discrimination as done here. In the current study, leakiness was neither correlated to *in vitro V*_pmax_/*V*_cmax_ nor to IS/CSR when all species and leaf temperatures were considered (Fig. 6). There were three exceptions at the species level: leakiness was positively correlated with *V*_pmax_/*V*_cmax_ (but not with IS/CSR) in *Ch. gayana, L. fusca*, and *Z. mays* (see Supplementary Figs S13 and S14). Hence, we argue that, in response to short-term changes in leaf temperature, CCM efficiency was balanced by biochemical factors, such as enzyme activity, as well as by physical factors, such as *g*_bs_ (von Caemmerer, 2000). A contribution by *g*_m_ is unlikely because very little influence of *g*_m_ on the carbon isotope discrimination (∆) has been predicted (von Caemmerer *et al.*, 2014).

### Conclusions

Modelling thermal photosynthetic responses at the leaf level is critical for predicting canopy-scale gas exchange in response to diurnal and seasonal changes in leaf temperature (Harley and Baldocchi, 1995). Thermal sensitivities of parameters used by the C_4_ photosynthesis model are needed to accurately predict CO_2_ exchange in response to temperature. The findings from the current study demonstrated that, like C_3_ photosynthesis (Bernacchi *et al.*, 2001; Walker *et al.*, 2013), *in vivo* and *in vitro* thermal responses of key photosynthetic parameters (e.g. PEPC and Rubisco activities) differ across C_4_ species. Hence, relying on the thermal responses of selected species to model C_4_ photosynthesis cannot accurately describe ecosystem responses.

The current study also demonstrated that variations in the thermal responses of leakiness (ϕ) among the C_4_ grasses is not aligned with the C_4_ subtypes. This indicates that various biochemical and anatomical trade-offs operate to maintain similar CCM efficiencies in the various C_4_ biochemical pathways. In addition, no correlations were observed among leakiness, *V*_pmax_/*V*_cmax_, and IS/CSR across a range of C_4_ grasses and leaf temperatures. Hence, more work is needed to characterize the thermal responses of *g*_m_ and *g*_bs_ in diverse C_4_ species by combining stable isotope and chlorophyll fluorescence studies (Yin *et al.*, 2016).

## Supplementary data

Supplementary data are available at *JXB* online.

Table S1. Summary of leaf gas exchange parameters for eight C_4_ grasses.

Table S2. Summary of *A*-*C*_i_-derived parameters and enzyme activities for eight C_4_ grasses.

Table S3. Rubisco and protein content of leaves.

Fig. S1. Light environment in the glasshouse during plant growth.

Fig. S2. Comparison of the June *et al.* (2004) and modified Arrhenius models for temperature response.

Fig. S3. Principle component analysis plots for species of the NADP-ME subtype, and all three C_4_ subtypes.

Fig. S4. Photosynthetic CO_2_ response curves (*A*-*C*_i_) measured at four-leaf temperatures in eight C_4_ grasses.

Fig. S5. Thermal responses of the CO_2_ assimilation rate (*A*), CO_2_-saturated rate (CSR), and initial slope of the *A*-*C*_i_ curve (IS) in eight C_4_ grasses fitted using the June *et al.* (2004) model.

Fig. S6. Thermal responses of photosynthetic enzyme activities in eight C_4_ grasses fitted using the June *et al.* (2004) model.

Fig. S7. Thermal responses of the CO_2_ assimilation rate (*A*), CO_2_-saturated rate (CSR), and initial slope of the *A*-*C*_i_ curve (IS) in eight C_4_ grasses fitted using the modified Arrhenius model.

Fig. S8. Thermal responses of photosynthetic enzyme activities in eight C_4_ grasses fitted using the modified Arrhenius model.

Fig. S9. Photosynthetic carbon isotope discrimination, Δ, as a function of *C*_i_/*C*_a_ measured during the gas exchange for eight C_4_ grasses.

Fig. S10. Relationship between CO_2_ assimilation rates and stomatal conductance in eight C_4_ grasses.

Fig. S11. Thermal response of leakiness in eight C_4_ grasses.

Fig. S12. Relationship between measured and modelled PEPC activity at various leaf temperatures in *Z. mays*.

Fig.S13. Relationship between leakiness and ratio of PEPC to Rubisco activity in eight C_4_ grasses.

Fig. S14. Relationship between leakiness and ratio of initial slope of *A*-*C*_i_ (IS) to CO_2_-saturated rates in eight C_4_ grasses.

## Supplementary Material

supplementary Tables
