# Supplementary material for: Short-term thermal photosynthetic responses of C4 grasses are independent of the biochemical subtype
Source: J Exp Bot. 2017 Oct 16;68(20):5583–97. doi: 10.1093/jxb/erx350 (PMC5853683; doi:10.1093/jxb/erx350)
Supplement: supplementary Tables [file erx350_suppl_supplementary_tables_s1-s3_figures.pdf]

SUPPLEMENTARY DATA

Table S1. Summary of leaf gas exchange parameters for eight C_4 grasses.

Leaf gas exchange was measured at PPFD of 1800 μmol m^{-2} s^{-1} and reference CO_2 of 400 μl L^{-1}. Values are means of 3-4 replicates ± SE. Letters indicate the ranking for temperature steps within each species using a multiple-comparison Tukey's Post Hoc test. Values followed by the same letter are not significantly different at 5% level. *P* values show significance levels derived by fitting linear model for all the C_4 species and linear mixed effect model for three C_4 subtypes at each single temperature. Significance levels are ns, not significant ($P > 0.05$); * $P< 0.05$; ** $P < 0.01$; *** $P < 0.001$.

**Parameater**	**Temp**	**NADP-ME**			**PEP-CK**			**NAD-ME**		**Subtype**			***P***	
	**(°C)**	***C. ciliaris***	***S. bicolor***	***Z. mays***	***M. maximus***	***C. gayana***	***E. meyeriana***	***P.coloratum***	***L. fusca***	**NADP-ME**	**PEP-CK**	**NAD-ME**	***Species***	***Subtype***
CO_2 assimilation	18	17 ± 1a	18 ± 2a	24 ± 0a	18 ± 1a	19 ± 0a	16 ± 1a	13 ± 1a	16 ± 1a	19 ± 1a	18 ± 1a	14 ± 1a	***	ns
rate, *A*	25	29 ± 1b	29 ± 2b	36 ± 1b	28 ± 2b	29 ± 0b	24 ± 1b	22 ± 0b	25 ± 1b	31 ± 1b	27 ± 1b	23 ± 1b	***	*
(μmol $m^{-2}s^{-1}$)	34	36 ± 1c	40 ± 1c	43 ± 1c	42 ± 0c	37 ± 3 c	40 ± 2c	34 ± 2c	40 ± 2c	40 ± 1c	40 ± 1c	37 ± 2c	*	ns
	40	40 ± 1c	39 ± 1c	37 ± 3bc	37 ± 2c	36 ± 1bc	44 ± 1c	35 ± 1c	38 ± 2c	38 ± 1c	39 ± 1c	36 ± 1c	0.06	ns
Stomatal	18	0.12 ± 0.01a	0.18 ± 0.02 a	0.27 ± 0.02a	0.15 ± 0.02a	0.19 ± 0.02a	0.14 ± 0.01a	0.12 ± 0a	0.13 ± 0.01 a	0.19 ± 0.02 a	0.16 ± 0.01a	0.12 ± 0.01a	***	ns
conductance,	25	0.2 ± 0.01b	0.23 ± 0.02 ab	0.34 ± 0.01ab	0.21 ± 0.02a	0.22 ± 0.01a	0.15 ± 0.01a	0.15 ± 0.01a	0.17 ± 0.01 a	0.24 ± 0.02 a	0.2 ± 0.01 a	0.16 ± 0.01b	***	ns
g_s (mol $m^{-2}s^{-1}$)	34	0.24 ± 0.01bc	0.33 ± 0.03bc	0.4 ± 0.01b	0.41 ± 0.01b	0.24 ± 0.02a	0.31 ± 0.02b	0.22 ± 0.01b	0.23 ± 0.01b	0.32 ± 0.03b	0.31 ± 0.02b	0.23 ± 0.01c	***	ns
	40	0.29 ± 0.02c	0.37 ± 0.03c	0.35 ± 0.03ab	0.36 ± 0.04b	0.24 ± 0.01a	0.38 ± 0.03b	0.26 ± 0.01b	0.27 ± 0.02 b	0.34 ± 0.02b	0.32 ± 0.03b	0.27 ± 0.01d	**	ns
VPD_L	18	1.2 ± 0.1 a	1 ± 0 a	1.1 ± 0.1a	1.2 ± 0.1 a	1.1 ± 0.1 a	1.2 ± 0 a	1.1 ± 0 a	1.2 ± 0.1 a	1.1 ± 0 a	1.2 ± 0 a	1.2 ± 0 a	ns	ns
(kPa)	25	1.6 ± 0.1 a	1.6 ± 0.2b	1.6 ± 0 b	1.4 ± 0.2 a	1.6 ± 0.1b	1.8 ± 0b	1.5 ± 0 a	1.7 ± 0 b	1.6 ± 0b	1.6 ± 0.1b	1.6 ± 0 a	ns	ns
	34	2.7 ± 0.3b	2.4 ± 0c	2.4 ± 0.1c	2.3 ± 0 b	2.6 ± 0c	2.7 ± 0.1c	2.4 ± 0.1b	3 ± 0.1c	2.5 ± 0.1c	2.5 ± 0.1c	2.7 ± 0.1b	ns	ns
	40	3.7 ± 0c	3.4 ± 0d	3.3 ± 0 d	3.7 ± 0.1c	3.9 ± 0.2d	3.5 ± 0.2d	3.3 ± 0.4b	4.1 ± 0.1d	3.5 ± 0.1d	3.7 ± 0.1d	3.6 ± 0.3c	0.06	ns
C_i/C_a	18	0.41 ± 0.02b	0.52 ± 0.02b	0.55 ± 0.02b	0.43 ± 0.05 a	0.54 ± 0.04c	0.46 ± 0 b	0.53 ± 0.05 b	0.48 ± 0.02c	0.5 ± 0.02 b	0.48 ± 0.02b	0.51 ± 0.03c	*	ns
	25	0.34 ± 0.02ab	0.36 ± 0.01a	0.42 ± 0.02a	0.34 ± 0.01 a	0.41 ± 0.01b	0.28 ± 0.01 a	0.33 ± 0.05 a	0.33 ± 0.03b	0.37 ± 0.01 a	0.36 ± 0.02 a	0.33 ± 0.03b	*	ns
	34	0.3 ± 0.02a	0.3 ± 0.04a	0.37 ± 0.02a	0.4 ± 0.01 a	0.34 ± 0.02ab	0.34 ± 0.02 a	0.27 ± 0.01 a	0.18 ± 0.01a	0.32 ± 0.02 a	0.36 ± 0.01 a	0.22 ± 0.02 a	**	**
	40	0.31 ± 0.02ab	0.37 ± 0.03a	0.39 ± 0.01a	0.4 ± 0.02 a	0.28 ± 0.0a	0.35 ± 0.03 a	0.35 ± 0.03 ab	0.3 ± 0.01b	0.35 ± 0.02 a	0.34 ± 0.02 a	0.33 ± 0.02b	*	ns
Photosynthetic	18	4.1 ± 0 a	2.2 ± 0a	3.5 ± 0.2b	2.7 ± 0.1a	4.1 ± 0.1b	2.2 ± 0.1 a	3.9 ± 0.1 a	4.3 ± 0 c	3.1 ± 0.3 a	2.9 ± 0.3 a	4.1 ± 0.1b	***	ns
carbon isotope	25	3.9 ± 0.1a	3.1 ± 0.1b	2.6 ± 0.1a	2.8 ± 0 a	3.8 ± 0.1a	3.3 ± 0 b	3.6 ± 0.2 a	4 ± 0.1 bc	3.4 ± 0.2 a	3.4 ± 0.1 a	3.7 ± 0.1ab	***	ns
discrimination, Δ	34	3.7 ± 0 a	3.1 ± 0.2b	2.8 ± 0.1a	2.7 ± 0.1 a	3.7 ± 0.1a	2.8 ± 0 b	3.7 ± 0.1 a	3.9 ± 0 b	3.2 ± 0.2 a	3.2 ± 0.1 a	3.8 ± 0 ab	***	ns
(‰)	40	3.6 ± 0.3a	3 ± 0.1b	2.5 ± 0 a	2.6 ± 0.1 a	3.6 ± 0.1a	3 ± 0.1 b	3.5 ± 0.1 a	3.4 ± 0.2 a	3.1 ± 0.2 a	3.1 ± 0.2 a	3.4 ± 0.1 a	***	ns
Leakiness, ϕ	18	0.42 ± 0 c	0.26 ± 0.01 b	0.38 ± 0.01c	0.27 ± 0.02b	0.42 ± 0.01d	0.25 ± 0.01b	0.4 ± 0.01c	0.43 ± 0c	0.34 ± 0.02b	0.31 ± 0.03b	0.42 ± 0.01c	***	ns
	25	0.36 ± 0.01 bc	0.25 ± 0.01 b	0.24 ± 0 b	0.21 ± 0.01a	0.35 ± 0.01c	0.25 ± 0b	0.31 ± 0.01b	0.37 ± 0.02b	0.31 ± 0.02b	0.28 ± 0.02ab	0.33 ± 0.01b	***	ns
	34	0.29 ± 0 ab	0.19 ± 0.01 a	0.21 ± 0 ab	0.2 ± 0 a	0.28 ± 0.01b	0.19 ± 0.01a	0.27 ± 0.01a	0.23 ± 0.01a	0.23 ± 0.01a	0.23 ± 0.02a	0.24 ± 0.01a	***	ns
	40	0.26 ± 0.06 a	0.2 ± 0 a	0.17 ± 0.01a	0.18 ± 0 a	0.24 ± 0.01a	0.2 ± 0.01ab	0.25 ± 0a	0.22 ± 0.01a	0.21 ± 0.02a	0.21 ± 0.01a	0.24 ± 0.01a	ns	ns
C_4 cycle rate, V_p	18	30 ± 1a	25 ± 3a	40 ± 1a	25 ± 2a	34 ± 1 a	23 ± 2 a	21 ± 2 a	28 ± 2 a	31 ± 2 a	27 ± 2 a	25 ± 2 a	***	ns
(μmol $m^{-2}s^{-1}$)	25	48 ± 3b	40 ± 2b	50 ± 1b	37 ± 3 b	47 ± 1 b	34 ± 1 b	34 ± 1 b	42 ± 2 b	46 ± 2 b	40 ± 2 b	37 ± 2 b	***	ns
	34	54 ± 1bc	52 ± 1c	58 ± 1c	56 ± 0 c	55 ± 5 b	53 ± 3 c	49 ± 3 c	54 ± 2 c	54 ± 1 c	55 ± 2 c	52 ± 2 c	ns	ns
	40	60 ± 3c	51 ± 1c	50 ± 3b	48 ± 2 c	52 ± 1 b	60 ± 2 c	50 ± 1 c	54 ± 4 c	53 ± 2 bc	53 ± 2 c	51 ± 2 c	**	ns
Dark respiration,	18	0.7 ± 0 a	0.5 ± 0 a	1.1 ± 0 a	0.3 ± 0 a	0.8 ± 0 a	0.9 ± 0.1 a	0.2 ± 0 a	0.4 ± 0 a	0.7 ± 0.1 a	0.7 ± 0.1 a	0.3 ± 0 a	***	ns
R_d	25	1.1 ± 0 b	0.8 ± 0.1b	1.6 ± 0 b	1.2 ± 0 b	1.2 ± 0 b	1.7 ± 0 b	1.1 ± 0 b	1.4 ± 0 b	1.2 ± 0.1 a	1.3 ± 0.1b	1.2 ± 0.1b	***	ns
(μmol m^{-2} s^{-1})	34	2.2 ± 0 c	1.3 ± 0 c	2.8 ± 0 c	2 ± 0 c	2 ± 0 c	3 ± 0 c	1.9 ± 0 c	1.8 ± 0 c	2.1 ± 0.2 b	2.3 ± 0.1c	1.8 ± 0 c	***	ns
	40	4.5 ± 0 d	1.9 ± 0 d	4 ± 0 d	2.2 ± 0.3c	3.8 ± 0 d	4.2 ± 0 d	2.8 ± 0 d	3.8 ± 0 d	3.4 ± 0.4 c	3.4 ± 0.3d	3.2 ± 0.3d	***	ns

Table S2. Summary of AC_i derived parameters and enzyme activities for eight C_4 grasses.

Values are means of 3-4 replicates ± SE. Letters indicate the ranking for temperature steps within each species using a multiple-comparison Tukey's Post Hoc test. Values followed by the same letter are not significantly different at 5% level. *P* values show significance levels derived by fitting linear model for all the C_4 species and linear mixed effect model for three C_4 subtypes at each single temperature. Significance levels are ns, not significant (*P* > 0.05); * *P* < 0.05; ** *P* < 0.01; *** P < 0.001.

Parameater	Temp	NADP-ME			PEP-CK			NAD-ME		Subtype			*P*	
		C. ciliaris	*S. bicolor*	*Z. mays*	*M. maximus*	*C. gayana*	*E. meyeriana*	*P.coloratum*	*L. fusca*	NADP-ME	PEP-CK	NAD-ME	*Species*	*Subtype*
Initial slope, IS	18	0.28 ± 0.05 a	0.1 ± 0.03 a	0.39 ± 0 a	0.25 ± 0.02 a	0.24 ± 0.03 a	0.22 ± 0.02 a	0.17 ± 0.03 a	0.29 ± 0.02 a	0.25 ± 0.05 a	0.23 ± 0.01 a	0.23 ± 0.03 a	***	ns
(µmol m^{-2} s^{-1}µ bar^{-1})	25	0.3 ± 0.03 a	0.33 ± 0.02 ab	0.65 ± 0.02b	0.33 ± 0.01 ab	0.46 ± 0.05b	0.29 ± 0 a	0.31 ± 0.02b	0.48 ± 0.02 ab	0.43 ± 0.06 a	0.36 ± 0.03 a	0.4 ± 0.04 a	***	ns
	34	0.55 ± 0.07b	0.58 ± 0.12 bc	0.68 ± 0.03b	0.41 ± 0.03b	0.6 ± 0.04b	0.49 ± 0.05b	0.45 ± 0.04 c	0.67 ± 0.05b	0.6 ± 0.05 a	0.5 ± 0.03 a	0.56 ± 0.06 a	*	ns
	40	0.77 ± 0.03c	0.77 ± 0.09c	0.62 ± 0.06b	0.41 ± 0.03b	0.59 ± 0.01b	0.54 ± 0.03b	0.49 ± 0.03c	0.71 ± 0.12b	0.72 ± 0.04 b	0.51 ± 0.03 a	0.6 ± 0.07 ab	**	0.06
CO_2 saturated rate,	18	20 ± 1 a	13 ± 1 a	23 ± 0 a	19 ± 2 a	19 ± 2 a	19 ± 1 a	16 ± 1 a	18 ± 1 a	19 ± 2 a	19 ± 1 a	17 ± 1 a	***	ns
CSR	25	33 ± 1 b	34 ± 0 b	33 ± 1 b	27 ± 0b	34 ± 3b	34 ± 1b	33 ± 1b	32 ± 1b	34 ± 1b	32 ± 1b	33 ± 1b	*	ns
(µmol m^{-2} s^{-1})	34	45 ± 2 c	47 ± 1 c	46 ± 1c	42 ± 2c	43 ± 0c	48 ± 3c	40 ± 1c	41 ± 1c	46 ± 1c	45 ± 1c	41 ± 1c	*	*
	40	50 ± 0 c	49 ± 1 c	34 ± 2b	38 ± 1c	43 ± 2c	46 ± 0c	39 ± 0 c	42 ± 1c	44 ± 3c	43 ± 1c	40 ± 1c	***	ns
IS/CSR	18	0.014 ± 0.002a	0.007 ± 0.001a	0.016 ± 0 a	0.013 ± 0 a	0.013 ± 0.002a	0.011 ± 0.001a	0.011 ± 0.002a	0.016 ± 0 a	0.012 ± 0.002a	0.012 ± 0.001a	0.013 ± 0.001a	**	ns
	25	0.009 ± 0.001a	0.01 ± 0.001ab	0.02 ± 0.001a	0.012 ± 0 a	0.014 ± 0.001a	0.009 ± 0a	0.009 ± 0.001a	0.015 ± 0.001a	0.013 ± 0.002a	0.011 ± 0.001a	0.012 ± 0.001a	***	ns
	34	0.013 ± 0.002a	0.012 ± 0.002ab	0.015 ± 0 a	0.01 ± 0.001 a	0.014 ± 0.001a	0.01 ± 0.001a	0.011 ± 0.001a	0.017 ± 0.002a	0.013 ± 0.001a	0.011 ± 0.001a	0.014 ± 0.001a	0.07	ns
	40	0.015 ± 0 a	0.016 ± 0.002b	0.018 ± 0.003a	0.011 ± 0.001a	0.014 ± 0.001a	0.012 ± 0.001a	0.013 ± 0.001a	0.017 ± 0.002a	0.017 ± 0.001b	0.012 ± 0.001a	0.015 ± 0.001ab	*	*
PEPC activity, V_{pmax}	18	46 ± 5 a	111 ± 3 a	84 ± 6 a	28 ± 11 a	49 ± 7 a	85 ± 9 a	19 ± 2a	19 ± 3 a	83 ± 9 a	54 ± 10 a	19 ± 2 a	***	*
(µmol m^{-2} s^{-1})	25	69 ± 9 a	190 ± 16 ab	166 ± 9 b	47 ± 19 a	86 ± 12 ab	148 ± 10 b	47 ± 4 ab	37 ± 7 a	139 ± 18 ab	94 ± 16 ab	42 ± 4 ab	***	ns
	34	110 ± 8 b	230 ± 25 b	234 ± 18 b	64 ± 27 a	127 ± 21b	205 ± 14 c	76 ± 5bc	53 ± 11 a	195 ± 21b	132 ± 23 ab	63 ± 8 b	***	0.08
	40	140 ± 7 b	193 ± 37 ab	199 ± 26 b	70 ± 30 a	136 ± 22b	253 ± 16c	85 ± 11c	53 ± 12 a	179 ± 17 b	153 ± 29 b	67 ± 10 b	***	ns
Rubisco activity, V_{cmax}	18	7 ± 1 a	17 ± 2 a	15 ± 3 a	12 ± 1 a	11 ± 2 a	10 ± 1 a	11 ± 0 a	9 ± 1 a	13 ± 2 a	11 ± 1 a	10 ± 1 a	**	ns
(µmol m^{-2} s^{-1})	25	16 ± 3 ab	30 ± 3 b	30 ± 1b	21 ± 1b	24 ± 3b	17 ± 2 ab	20 ± 1ab	18 ± 1b	26 ± 3b	21 ± 1b	19 ± 1b	***	ns
	34	20 ± 4 ab	37 ± 2 bc	45 ± 1c	30 ± 2c	35 ± 3bc	25 ± 2bc	29 ± 2bc	28 ± 3c	34 ± 4bc	30 ± 2 c	29 ± 2c	***	ns
	40	23 ± 4 b	45 ± 2 c	49 ± 2c	35 ± 2c	41 ± 3c	29 ± 3c	32 ± 5 c	35 ± 3c	40 ± 4 c	35 ± 2 c	34 ± 3c	***	ns
PEPC/Rubisco	18	6.3 ± 0.9 a	6.8 ± 0.7 b	6.4 ± 1.9 a	2.1 ± 0.7 a	4.6 ± 0.3 a	8.3 ± 0.5 a	1.7 ± 0.2 a	2.3 ± 0.4a	6.6 ± 0.6 b	5 ± 0.9 a	2 ± 0.2 a	***	*
activty, V_{pamax}/V_{cmax}	25	5.2 ± 0.9 a	6.3 ± 0.2 ab	5.5 ± 0.3 a	2.2 ± 0.8 a	3.6 ± 0.1 a	8.7 ± 0.7 a	2.4 ± 0.2 ab	2 ± 0.2 a	5.7 ± 0.3ab	4.8 ± 1 a	2.2 ± 0.2a	***	ns
	34	5.8 ± 0.8 a	6.2 ± 0.4 ab	5.2 ± 0.3 a	2.1 ± 0.8 a	3.6 ± 0.3 a	8.3 ± 0.8 a	2.6 ± 0.1b	1.9 ± 0.2a	5.8 ± 0.3ab	4.7 ± 1 a	2.2 ± 0.2a	***	ns
	40	6.4 ± 0.8 a	4.2 ± 0.6 a	4 ± 0.4 a	2 ± 0.7 a	3.3 ± 0.5 a	8.9 ± 1.1 a	2.7 ± 0.3b	1.5 ± 0.2a	4.8 ± 0.5a	4.7 ± 1.1a	2 ± 0.3a	***	ns

Table S3. Rubisco and protein content.

Rubisco content, Rubisco activation, protein contents and % Rubisco of protein were measured in the leaves of eight C_4 grasses. Leaf sample were harvested during the sunny midday. Values are means (n = 3-4) ± SE. The effect of species was compared using linear model with type II ANOVA. The effect of subtype was compared using linear mixed effect model with type II ANOVA, where species were treated as a random variable. Letters indicate the ranking of species within each single row derived using a multiple-comparison Tukey's Post Hoc test. Values followed by the same letter are not significantly different at 5% level.

**Species/ subtype**	**Rubisco content (sites/m^2)**	**Rubisco activation (%)**	**Protein content (g/m^2)**	**% Rubisco of protein (w/w)**
C. ciliaris	4 ± 0.3a	70 ± 8 b	6.6 ± 2a	5.6 ± 1.5a
S. bicolor	5.8 ± 0.5ab	72 ± 6 b	6 ± 0.6ab	6.5 ± 0.3a
Z. mays	7.2 ± 0.4 b	63 ± 7ab	5.8 ± 0.7 b	8.6 ± 1.2a
M. maximus	5.7 ± 0.7ab	54 ± 5ab	5.4 ± 1.2ab	7.1 ± 1.3a
C. gayana	5.5 ± 0.2ab	62 ± 5ab	6.5 ± 1.5ab	6 ± 1.8a
E. meyeriana	3.8 ± 0.4a	41 ± 7a	5.4 ± 0.6a	5.1 ± 1a
P. coloratum	7.8 ± 0.5 b	54 ± 8ab	6.9 ± 2.2 b	8.9 ± 2.2a
L. fusca	4 ± 0.5a	54 ± 4ab	6.6 ± 1.5a	4.8 ± 1.1a
NADP-ME	5.4 ± 0.4a	69 ± 4 b	6.2 ± 0.8a	6.7 ± 0.8a
PEP-CK	5 ± 0.4a	53 ± 4a	5.7 ± 0.6a	6.1 ± 0.8a
NAD-ME	5.7 ± 0.8a	54 ± 4ab	6.7 ± 1.2a	6.5 ± 1.3a

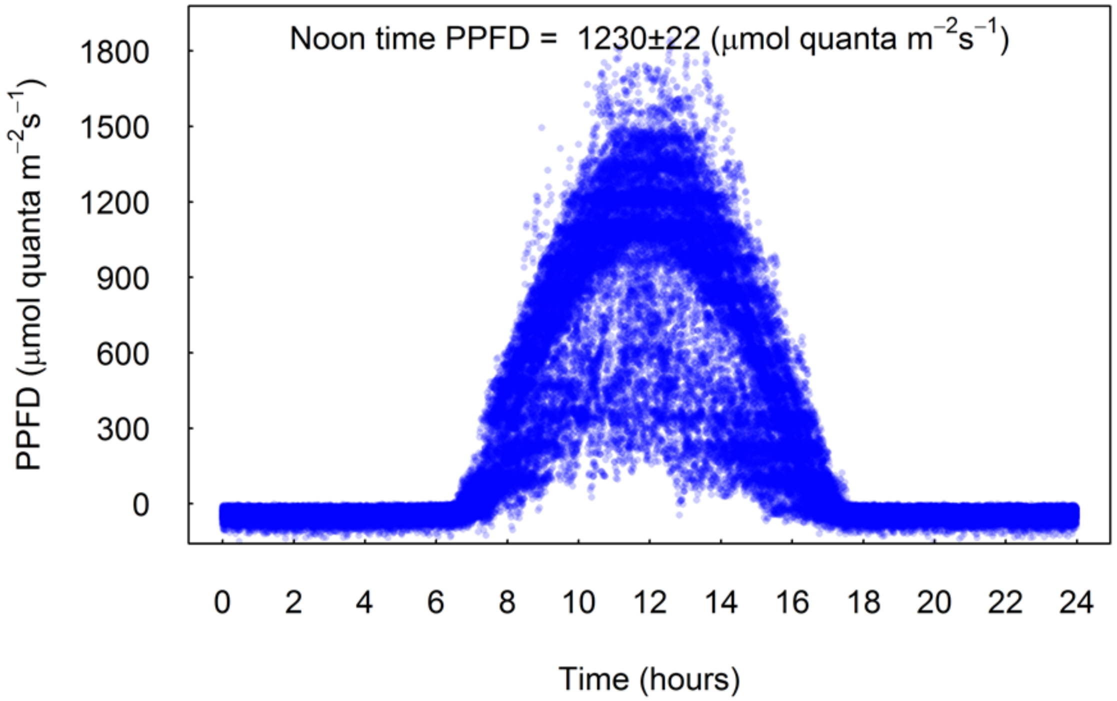

Fig. S1. Light environment in the glasshouse during plant growth.

Ground level data was collected from a nearby (2 km away) weather station. Glasshouse light level was calculated considering 80% light transmission through the glass material. Natural light duration was about 10 hr with an average PPFD = 1230 ± 22 µmol quant m^{-2} s^{-2} at noon.

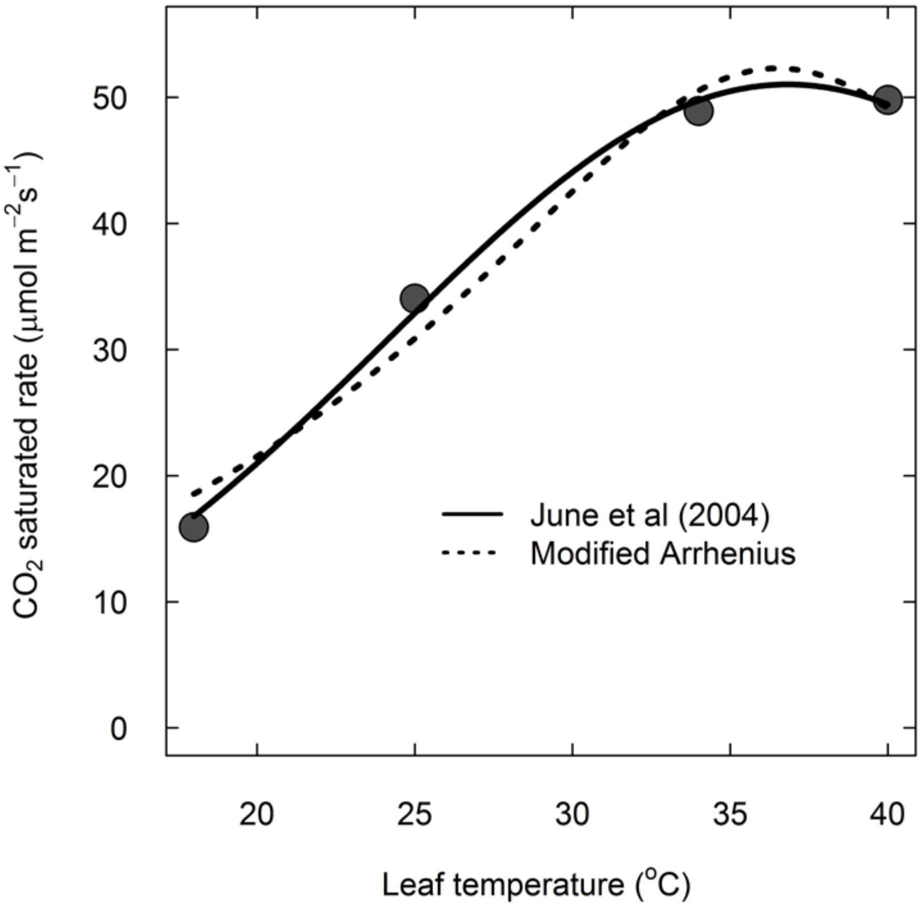

Fig. S2. Comparison of June *et al.* (2004) and modified Arrhenius model for temperature response.

Data points represent temperature response of CO_2 saturated rate (CSR) in *Sorghum bicolor*. The modified Arrhenius equation (Eqt 10) predicted (dotted line) higher temperature optimum (T_{opt}) and hence optimum CSR at T_{opt} than the June *et al.* (2004) equation (Eqt 11).

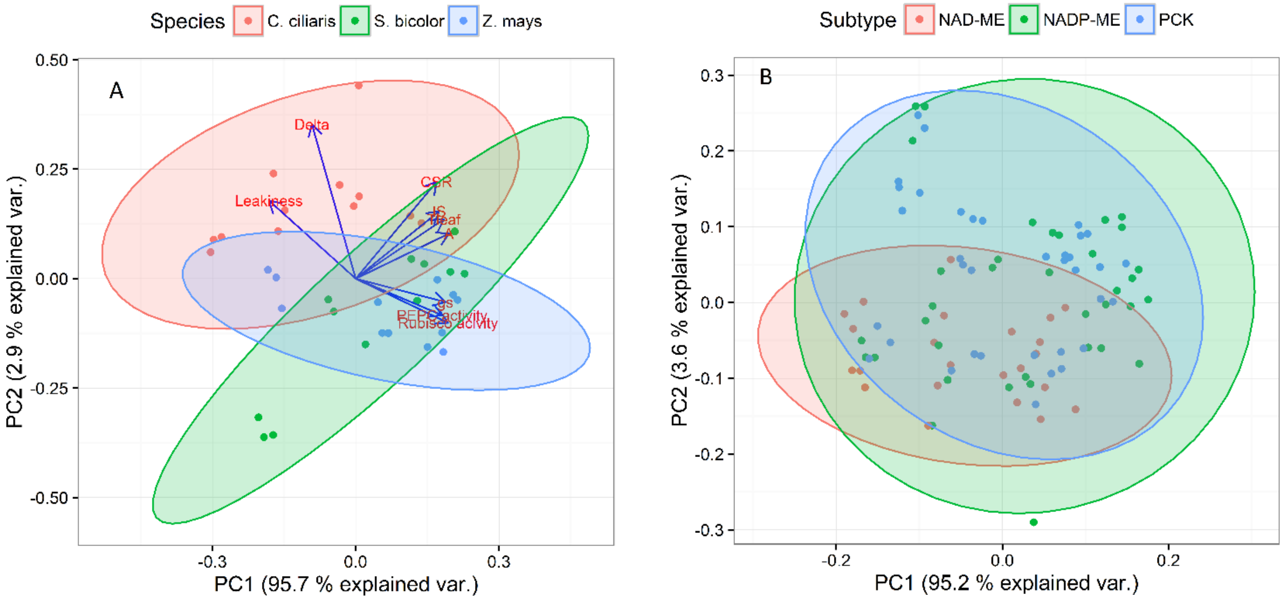

Fig. S3. Principle component analysis bi-plot for (A) species of the NADP-ME subtype or (B) the three C_4 subtypes.

Principle component analysis (PCA) performed on leaf gas exchange and enzyme activity parameters measured at 18, 25, 32 and 40°C for eight C_4 grasses belonging to three biochemical subtypes. Shaded area represents ellipse for each species assuming normal distribution of variables.

Parameters included in the PCA: CO_2 assimilation rates, stomatal conductance, C isotope discrimination, leakiness, initial slope of A-C_i curves, CO_2 saturated rates, Rubisco activity and PEPC activity.

PEP-CK activity for the three NADP-ME species are shown below. Enzyme activity was measured at 25°C in plants grown at high light and 28°C and receiving ample water and nutrition. Data are taken from a different experiment.

Z. mays 25 µmol m^{-2} s^{-1}

C. ciliaris 16 µmol m^{-2} s^{-1}

S. bicolor 3 µmol m^{-2} s^{-1}

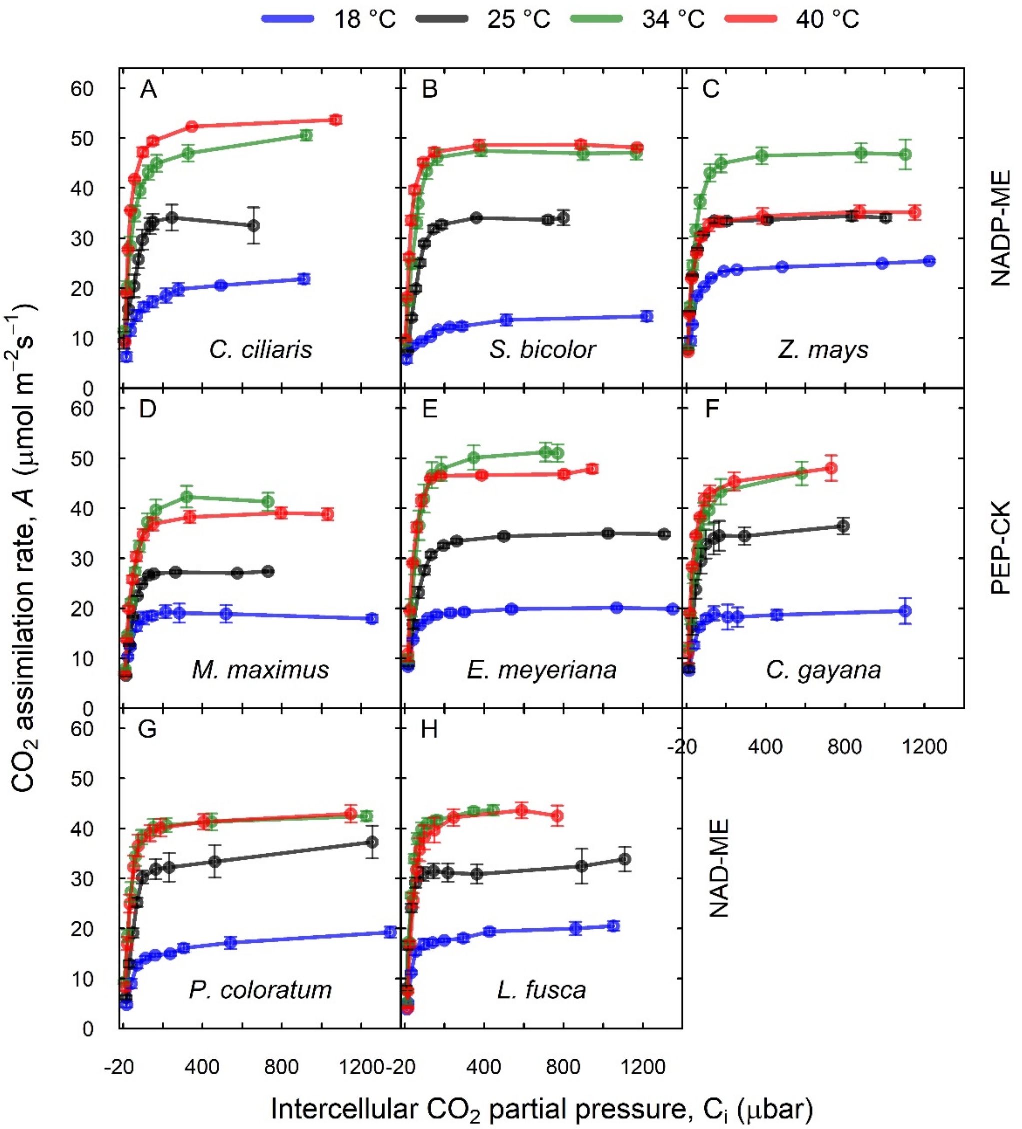

Fig. S4. Photosynthetic CO_2 response curves (A-C_i) measured at four-leaf temperature in eight grasses.

Responses of CO_2 assimilation rate to increasing intracellular [CO_2] were measured at 18 (dotted line), 25 (dashed line), 34 (long-dashed line) and 40°C (continuous line) in C_4-NADP-ME (A-C), C_4-PEP-CK (D-F) and C_4-NAD-ME (G-H) grasses. Leaves were measured at 1800 µmol m^{-2} s^{-1} PPFD. Values are means of 3-4 replicates ± SE.

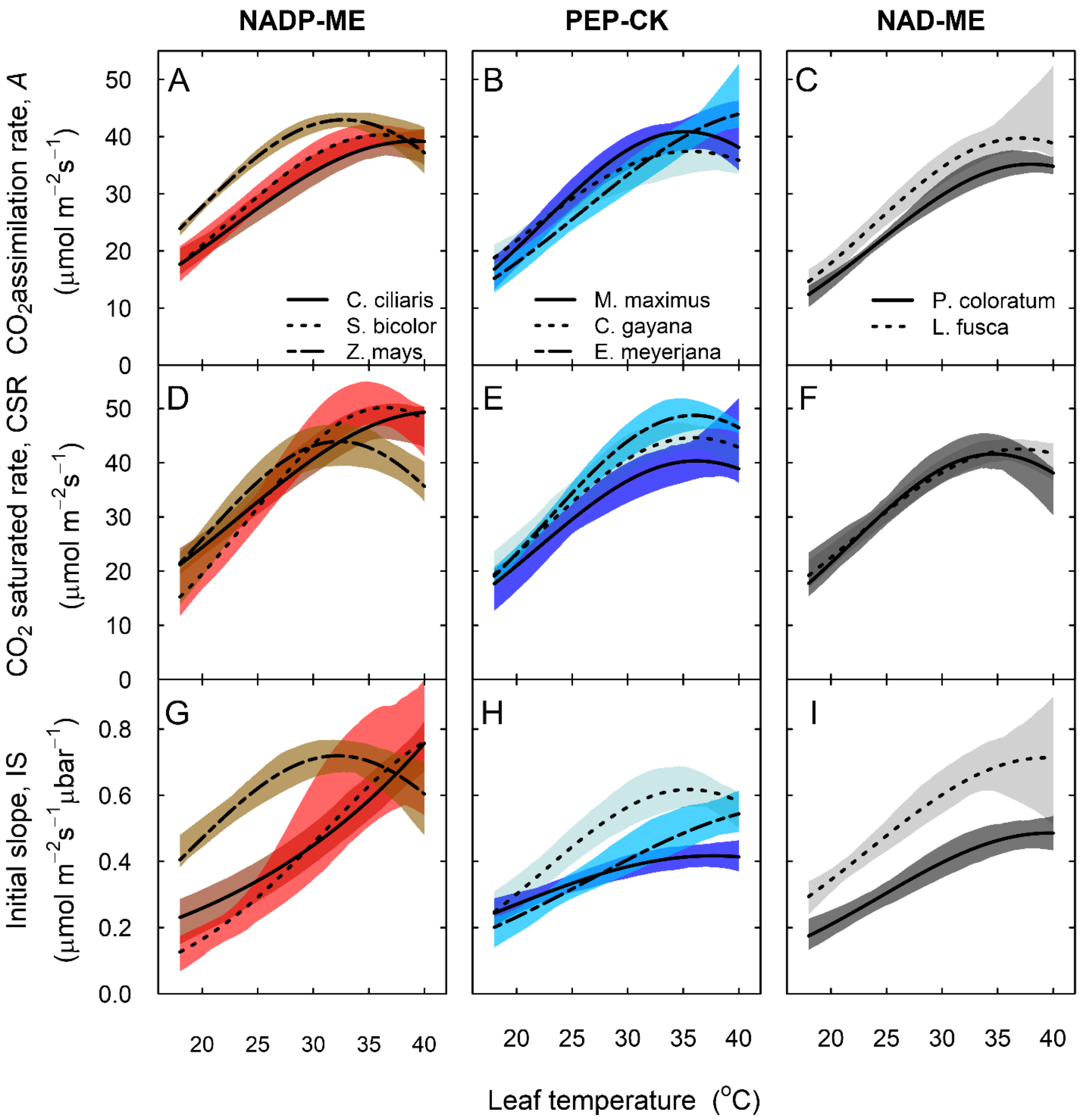

Fig. S5. Thermal responses of the CO_2 assimilation rate (*A*), CO_2 saturated rate (CSR) and initial slope of the AC_i curve (IS) in eight C_4 grasses fitted using the June *et al.* (2004) model.

CO_2 assimilation rate, *A* (A- C), CO_2 saturated rate, CSR (D-F), initial slope of CO_2 response curve, IS (G-I) as a function of leaf temperature for C_4-NADP-ME (A, D, G), C_4-PEP-CK (B, E, H) and C_4-NAD-ME (C, F, I) grasses. Shaded areas represent approx. 95% CI for the mean as estimated with the bootstrap fitted according to June *et al.* (2004) model (Eqt 11) and the derived parameters are shown in Table 2. Leaves were measured at 1800 µmol m^{-2} s^{-1}. Values are means of 3-4 replicates ± SE.

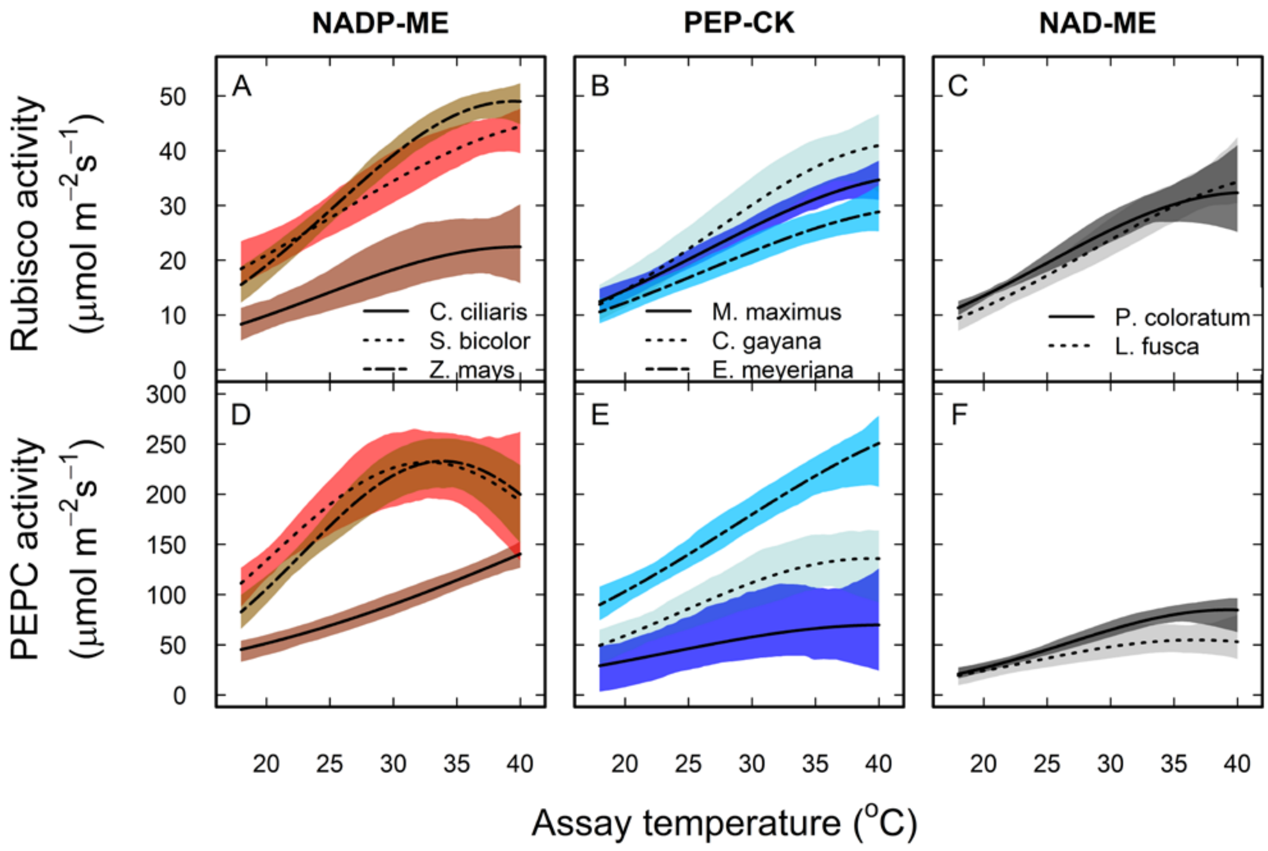

Fig. S6. Thermal responses of photosynthetic enzyme activities in eight C_4 grasses fitted using June et al. (2004) model.

Rubisco activity (A-C) and PEPC activity (D-F) as a function of temperature for C_4-NADP-ME (A, D, G), C_4-PEP-CK (B, E, H) and C_4-NAD-ME (C, F, I) grasses. For each extract, the temperature responses of both enzymes were measured at 18, 25, 34, and 40 °C. Shaded areas represent approx. 95% CI for the mean as estimated with the bootstrap fitted according to June *et al.* (2004) model (Eqt 11) and the derived parameters are shown in Table 2. Values are means of 3-4 replicates ± SE

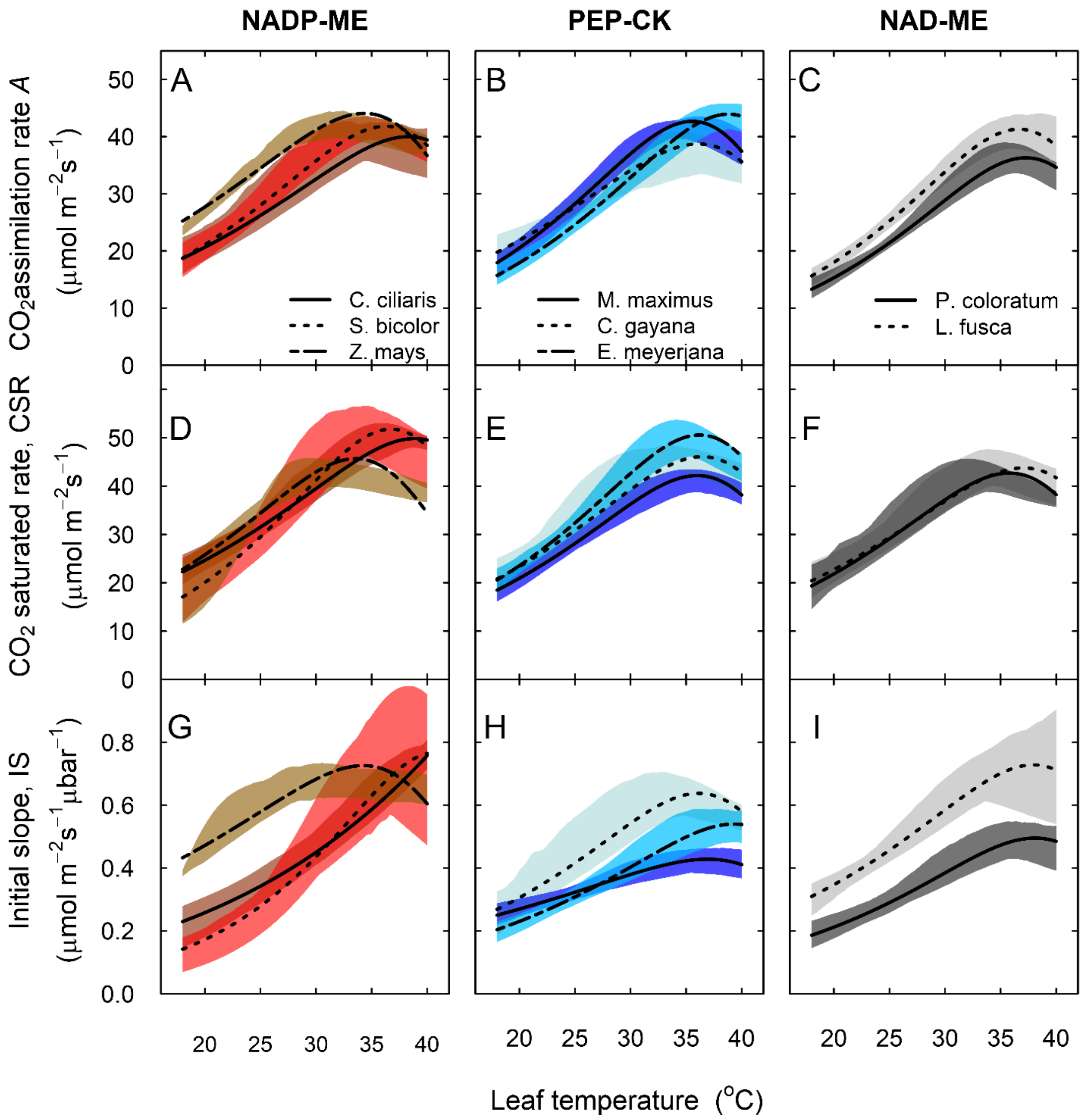

Fig. S7. Thermal responses of the CO_2 assimilation rate (*A*), CO_2 saturated rate (CSR) and initial slope of the AC_i curve (IS) in eight C_4 grasses fitted using modified Arrhenius model.

CO_2 assimilation rate, *A* (A- C), CO_2 saturated rate, CSR (D-F) and initial slope of CO_2 response curve, IS (G-I) as a function of leaf temperature for C_4-NADP-ME (A, D, G), C_4-PEP-CK (B, E, H) and C_4-NAD-ME (C, F, I) grasses. Shaded areas represent approx. 95% CI for the mean as estimated with the bootstrap fitted according to the modified Arrhenius model (Eqt 10) and the derived parameters are shown in Table 2. Leaves were measured at 1800 µmol m^{-2} s^{-1}. Values are means of 3-4 replicates ± SE.

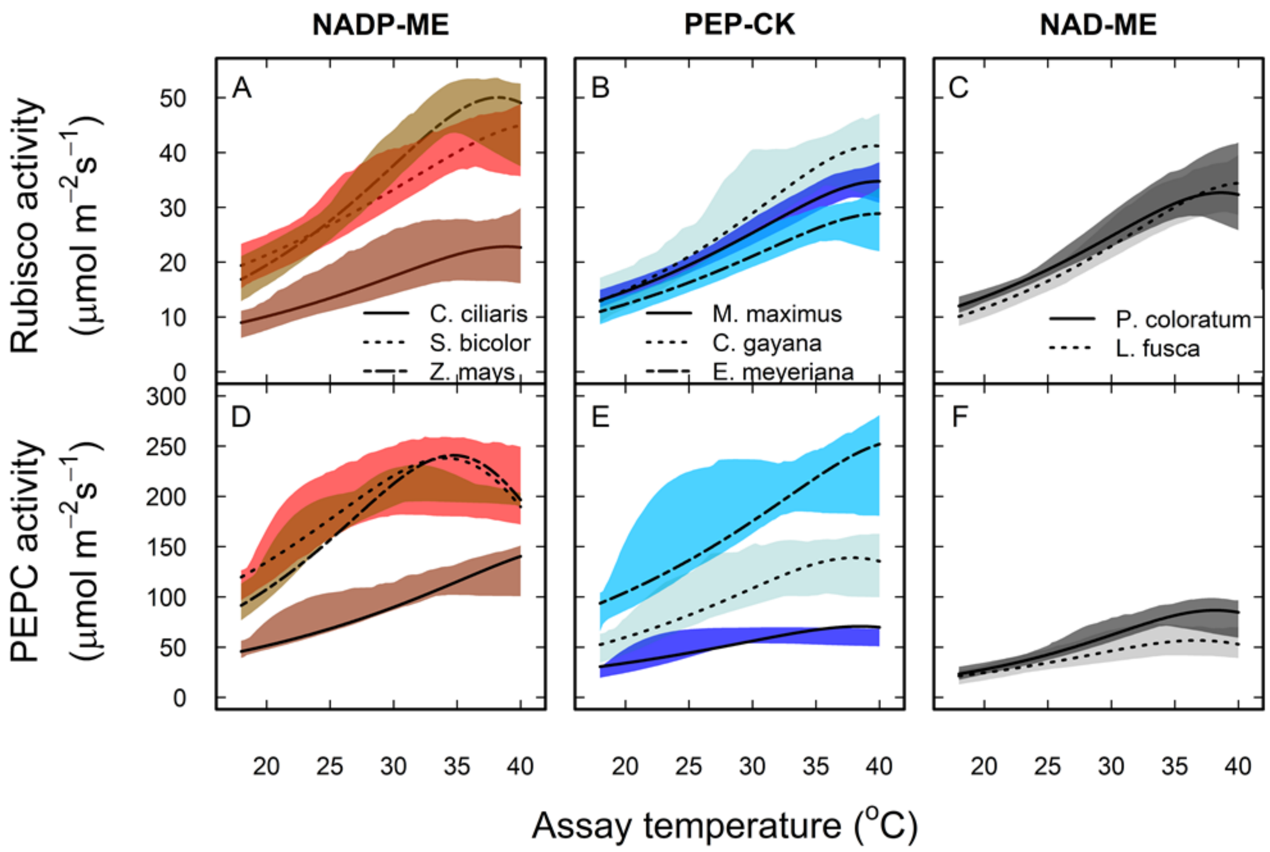

Fig. S8. Thermal responses of photosynthetic enzyme activities in eight C_4 grasses fitted using modified Arrhenius model.

Rubisco activity (A-C) and PEPC activity (D-F) as a function of temperature for C_4-NADP-ME (A, D, G), C_4-PEP-CK (B, E, H) and C_4-NAD-ME (C, F, I) grasses. For each extract, the temperature responses of both enzymes were measured at 18, 25, 34, and 40 °C. Shaded areas represent approx. 95% CI for the mean as estimated with the bootstrap fitted according to the modified Arrhenius model (Eqt 10) and the derived parameters are shown in Table 2. Values are means of 3-4 replicates ± SE.

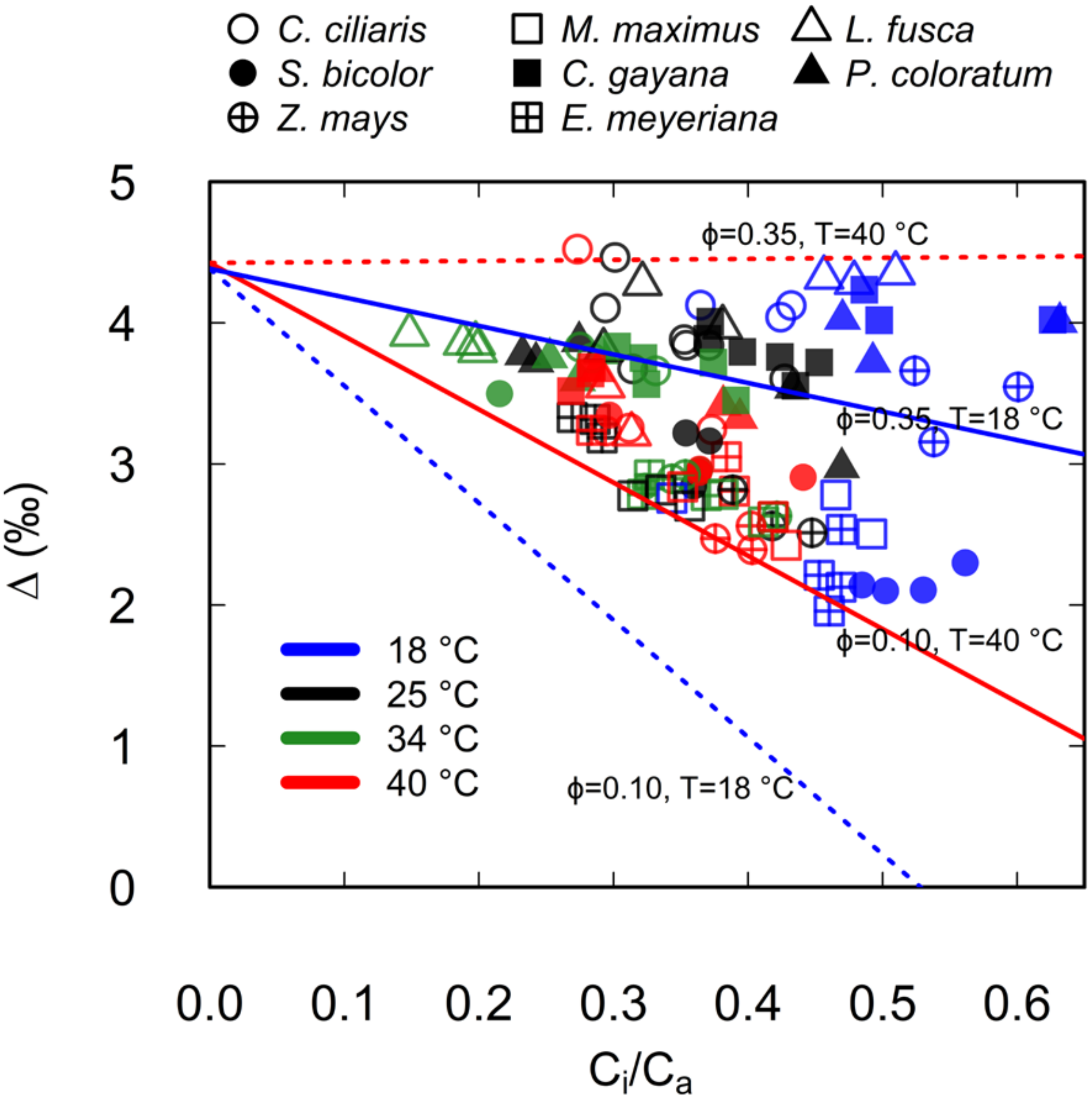

Fig. S9. Photosynthetic carbon isotope discrimination, Δ as a function of C_i/C_a measured during the gas exchange for eight C_4 grasses.

Lines are a solution of the updated C_4 discrimination model and includes the ternary formulation suggested by Farquhar and Cernusak (2012) and Pengelly *et al.* (2012) using leakiness ϕ = 0.35 at T = 18 °C (blue-solid line) or T = 40 °C (red-dotted line) and ϕ = 0.10 at T = 18 °C (blue-dotted line) or T = 40 °C (red-solid line). Leaf gas exchange was measured at high light (1800 μmol m^{-2} s^{-1}), ambient CO_2 (400 μL L^{-1}) and varying leaf temperature (18, 25, 34 and 40 °C). The symbols represent C_4 grasses belonging to the C_4-NADP-ME (circle), C_4-PEP-CK (squares) and C_4-NAD-ME (triangles) subtypes.

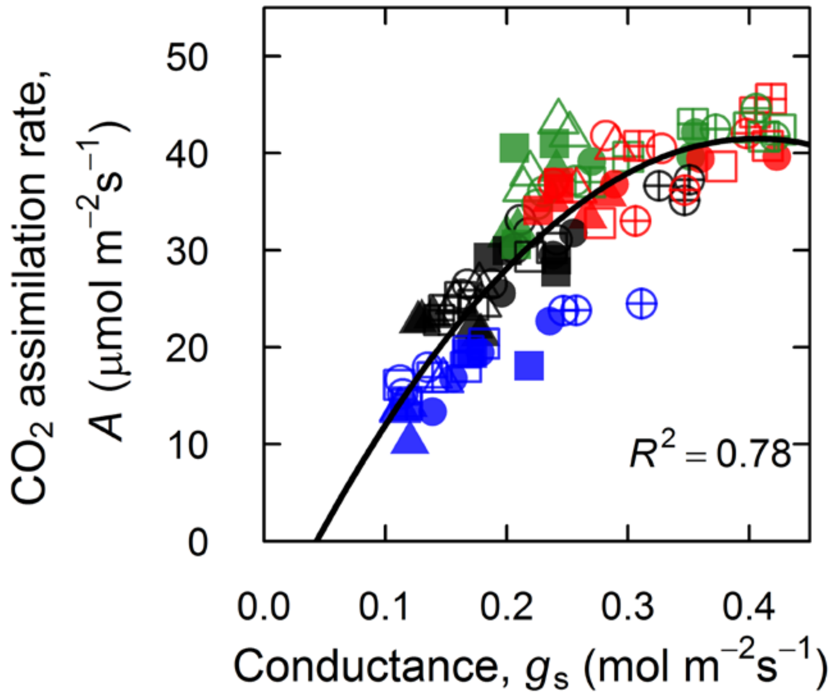

Fig. S10. Relationship between CO_2 assimilation rates and stomatal conductance in eight C_4 grasses.

Relationship between CO_2 assimilation rates and stomatal conductance in eight C_4 grasses measured at 18, 25, 34 and 40 °C. Solid line represent regression of all data points ($y = 314.7x^2 + 255.7x - 10.45$). Symbols are similar to those used in S2.

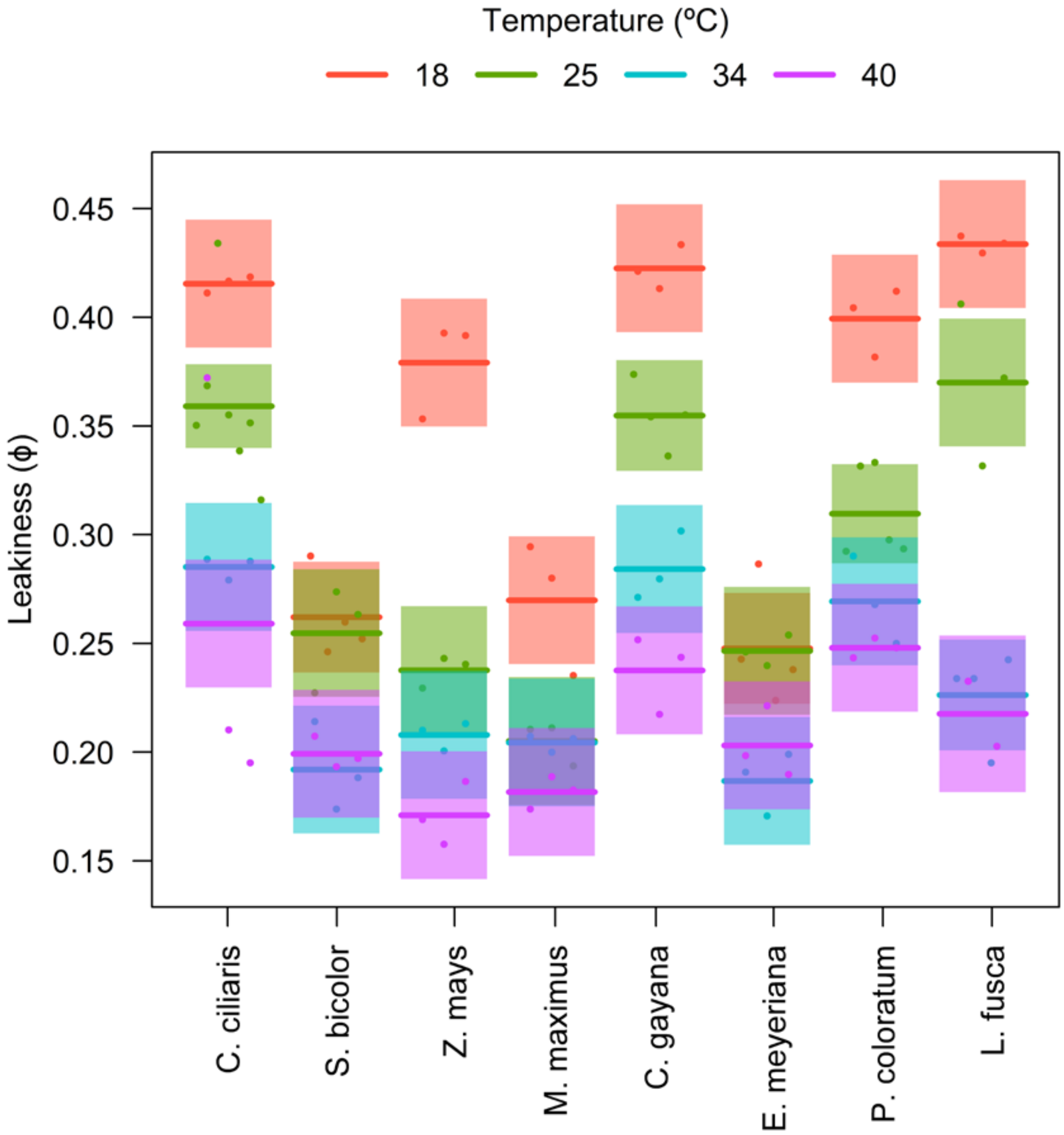

825

826 **Fig. S11. Thermal response of leakiness in C_4 grasses.**

827 Calculated leakiness at four temperatures (18, 25, 34 and 40 °C) in eight C_4 grasses. Shaded portion

828 indicated 95% confidence intervals.

829

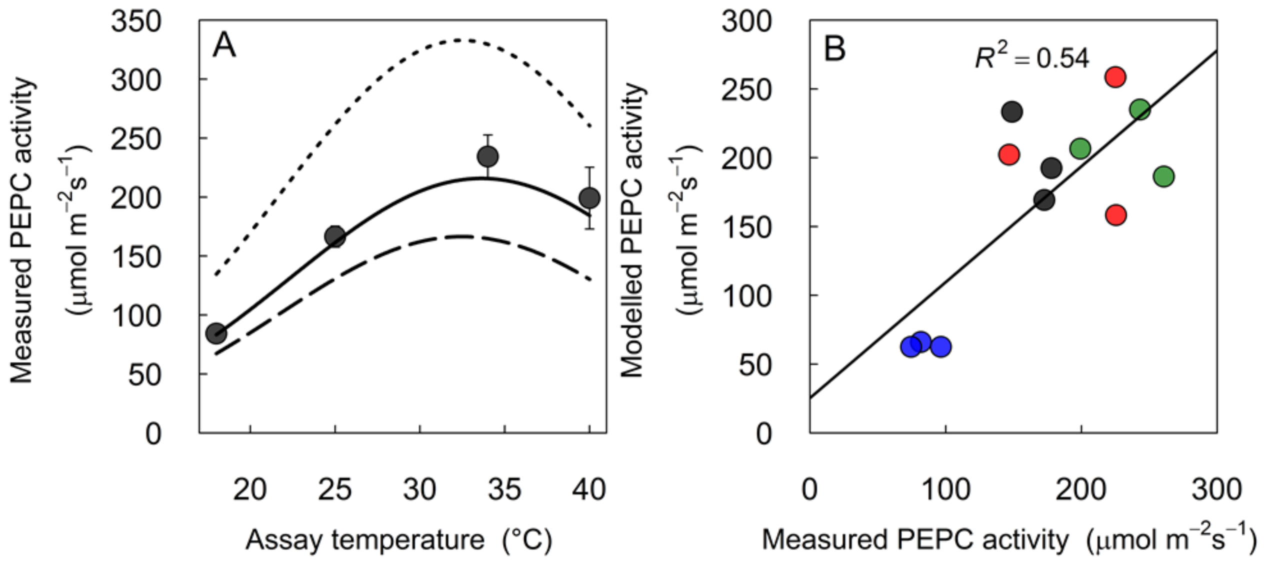

Fig. S12. Relationship between measured and modelled PEPC activity at various leaf temperature in *Z. mays.*

PEPC activity was measured by an *in vitro* assay at 18, 25, 34 and 40 °C (circles). To model PEPC activity, the initial slopes (IS) of the AC$_i$ curves measured at 18, 25, 34 and 40 °C were used together with g_m, K_p and their respective activation energy determined for *Z. mays* by (Ubierna *et al.* 2016) and (Boyd *et al.* 2015) using the expression $PEPC\ activity = \frac{K_p \cdot IS \cdot g_m}{g_m - IS}$ according to (Pfeffer & Peisker 1998). A. Modeled PEPC activity for *Z. mays*; dotted line (….) with g_m = 1 mol m^{-2} s^{-1} (Ubierna *et al.* 2016) and K_p = 160 µbar (Boyd *et al.* 2015); dashed line (----) with g_m = 1 mol m^{-2} s^{-1} and K_p = 80 µbar (Bauwe 1986); and continuous line (—) with g_m = 1.1 mol m^{-2} s^{-1} and K_p = 115 µbar (both fitted values). B. The relationship between modelled and assayed PEPC activity for *Z. mays*. Circle represents measurement temperature: 18 (blue), 25 (black), 34 (green) and 40 °C (red).

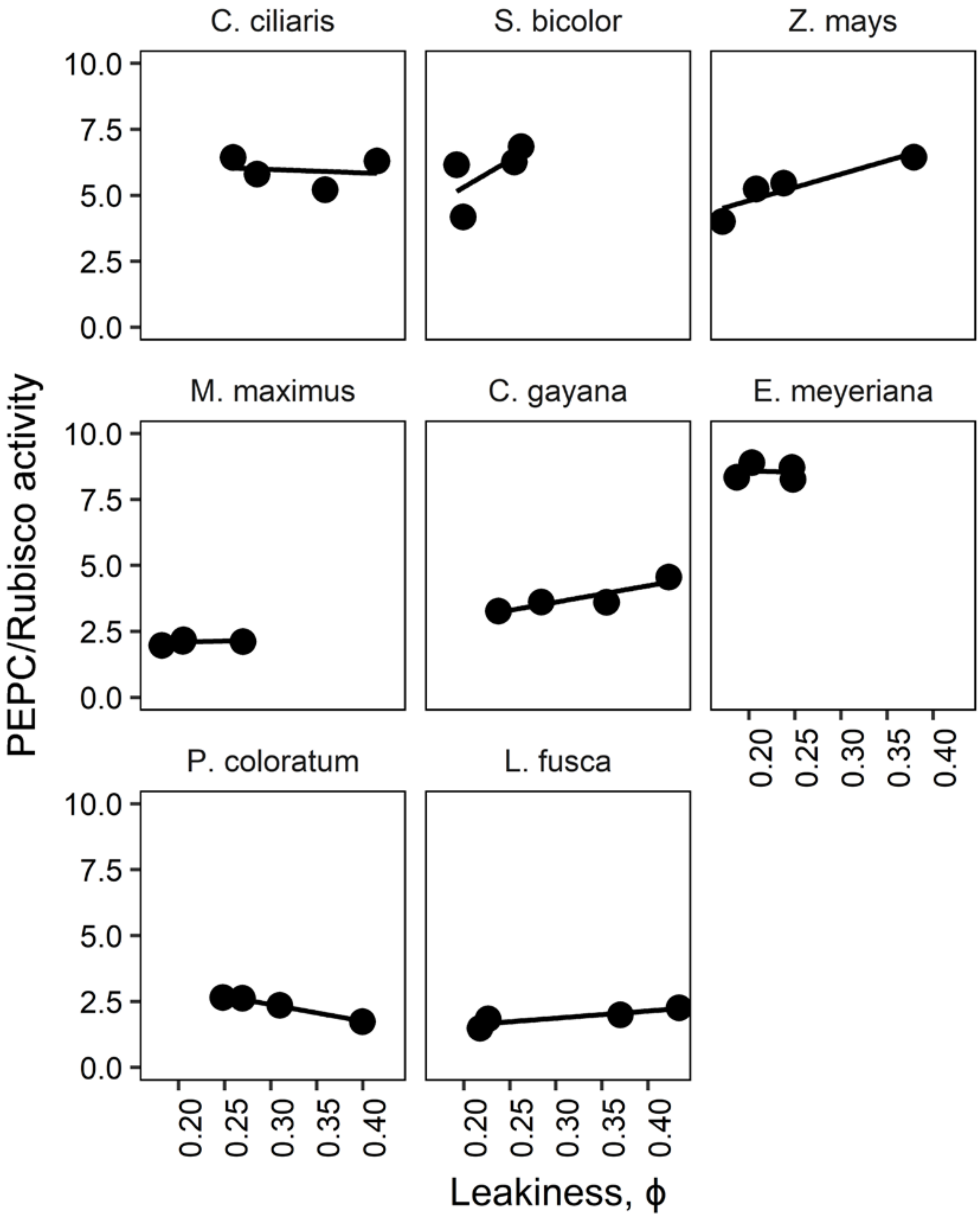

Fig.S13. Relationship between leakiness and ratio of PEPC to Rubisco activity in eight C_4 grasses.

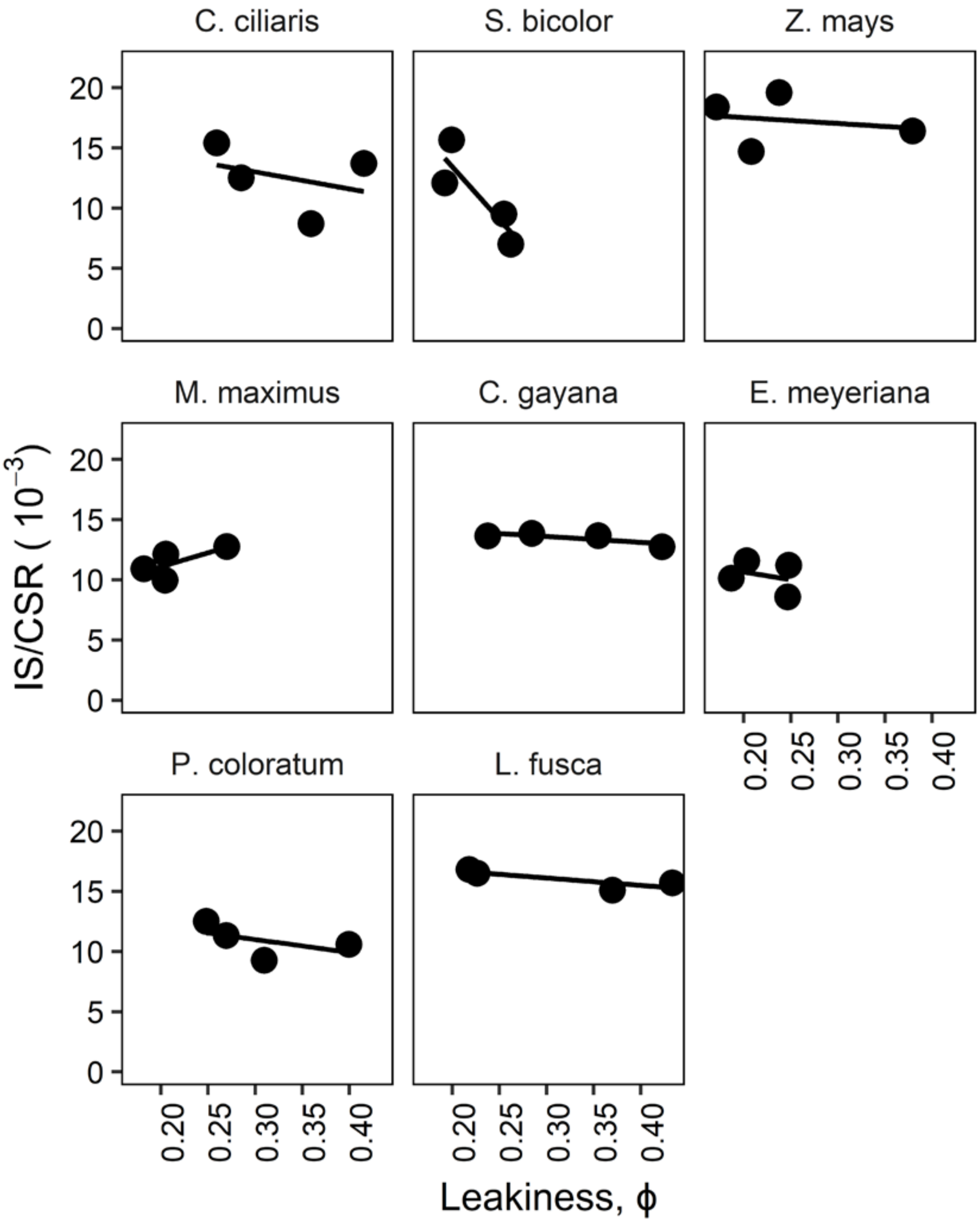

Fig. S14. Relationship between leakiness and ratio of initial slope of ACi (IS) to CO_2 saturated rates in eight C_4 grasses.
